# Learning-centred use of generative AI and later academic functioning: a baseline-adjusted three-wave panel study

**DOI:** 10.3389/fpsyg.2026.1878514

**Published:** 2026-07-01

**Authors:** Yang Zhao, Jian Chen, Wei Dai, Yuan Gu

**Affiliations:** 1 School of Law, Southwest University of Science and Technology, Mianyang, Sichuan, China; 2 Department of Geography, University College London, London, United Kingdom; 3 Research Institute of Science, Technology and Industrial Development, Sichuan University of Architectural Technology, Deyang, Sichuan, China; 4 Department of Public Administration and Humanities, Dalian Maritime University, Dalian, Liaoning, China

**Keywords:** academic procrastination, academic self-efficacy, generative artificial intelligence, higher education, learning engagement, learning-centred use, self-regulated learning

## Abstract

**Introduction:**

Generative artificial intelligence (GenAI) is rapidly entering higher education, but measures of adoption, frequency, intention, or general attitude do not explain whether students use GenAI in ways that support academic work. This study focused on learning-centred use of GenAI in academic work (LCU), defined as a task-focused, verification-oriented, and non-substitutive pattern of GenAI integration, and tested time-ordered paths from LCU to later academic functioning through self-regulated learning (SRL) and academic self-efficacy (ASE).

**Methods:**

We analysed a de-identified three-wave panel dataset from university students in China. The valid baseline panel included 1,200 students; 984 students were retained at T2, where SRL and ASE were measured in separated modules (T2a and T2b); and 788 complete cases were retained at T3, where academic procrastination and learning engagement were assessed. Baseline-adjusted composite-score path models controlled for prior SRL, ASE, procrastination, engagement, demographic characteristics, and GenAI-use covariates. Inverse probability weighting and auxiliary behavioural and rater indicators were used as sensitivity and convergence checks.

**Results:**

T1 LCU positively predicted T2a SRL (*β* = 0.345, *p* < 0.001) and T2b ASE (*β* = 0.118, *p* = 0.002), and T2a SRL positively predicted T2b ASE (*β* = 0.244, *p* < 0.001). SRL and ASE subsequently predicted lower T3 academic procrastination and higher T3 learning engagement after baseline outcomes were controlled. Indirect effects from LCU to both T3 outcomes were supported through SRL, ASE, and the SRL-to-ASE sequence; no residual direct LCU-to-outcome paths remained statistically significant after these mechanisms were included. Inverse probability weighting (IPW) and auxiliary-indicator checks showed a similar substantive pattern.

**Discussion:**

These findings show that the academic value of GenAI use is realised when students integrate it into learning-centred regulatory practice. Compared with frequency-based accounts, the present model identifies LCU as the behavioural condition that connects GenAI use with later academic functioning through strengthened SRL and ASE. The study advances a psychologically grounded account of GenAI in higher education and highlights the need to guide students toward task-focused, verification-oriented, and non-substitutive use that supports planning, monitoring, persistence, and engagement.

## 1 Introduction

Students now encounter generative artificial intelligence (GenAI) during ordinary academic work. They may ask a chatbot to explain a difficult reading, compare solution routes, generate examples, debug code, or review a draft. These uses usually begin with a need for support: a task is unclear, feedback is unavailable, time is limited, or progress has stalled. What matters educationally is what students do next. Some students use GenAI to clarify the task, plan their next step, check their reasoning, and revise their own work. Others may move quickly from uncertainty to answer substitution. The same act of “using GenAI” can therefore involve very different forms of academic engagement.

A central limitation in the current literature is that student GenAI use is often measured through adoption, frequency, intention to use, perceived usefulness, or general attitude. These indicators are valuable for documenting diffusion and acceptance, but their explanatory value for academic functioning remains limited because the same reported frequency can involve different academic practices. One student may use GenAI to clarify concepts, identify a starting point, request hints after an impasse, verify claims, and revise a draft. Another may use it mainly to obtain a finished answer with little evaluation. The same category of ‘use’ can contain different regulatory, motivational, and judgement-related processes. For this reason, the present study focuses on the quality of academic use within learning activity.

This distinction is visible across recent GenAI education research. Some studies emphasise the instructional affordances of GenAI, including explanation, feedback, writing support, programming support, and task scaffolding (Kasneci et al., 2023; Yilmaz and Karaoglan Yilmaz, 2023; Baek et al., 2024; Chen and Cheung, 2025). Other studies emphasise risks such as shallow processing, overreliance, misinformation, and academic integrity problems (Kasneci et al., 2023; Yang et al., 2025). Recent mechanism-oriented studies have advanced the field by linking AI attitudes, GenAI-assisted learning, self-regulation, self-efficacy, motivation, engagement, and deep learning through mediation or structural models (Liang and Reiss, 2025; Liu et al., 2025; Dong, 2026; Shao and Wang, 2026; Wang, 2026; Xu, 2026). These advances leave a central question for educational psychology: whether students’ later academic functioning is associated with the quality of their academic use of GenAI, especially the ways they bring it into ongoing academic work.

International work has also shown that GenAI integration is shaped by institutional policy, cultural context, and technology-acceptance conditions. Studies of university policies across global regions and within the United States suggest that higher education institutions are moving toward cautious acceptance, usually combining opportunities for teaching and learning with concerns about academic integrity, accuracy, privacy, equitable access, and authentic assessment (Wang et al., 2024; Jin et al., 2025). A parallel body of student-focused research has used TAM, UTAUT, UTAUT2, or related acceptance frameworks to explain whether students intend to use, adopt, or continue using GenAI tools (Sergeeva et al., 2025; Sousa and Cardoso, 2025). These studies are useful for understanding diffusion and acceptance, but they do not by themselves show whether students use GenAI in ways that preserve academic judgement, checking, and revision. This is the point at which the present study departs from adoption-focused accounts.

The present study addresses this gap by focusing on learning-centred use of GenAI in academic work (LCU), a deliberate, task-focused, active, and non-substitutive pattern of use. In this pattern, students draw on GenAI to clarify task demands, organise a workable starting point, compare alternatives, obtain hints after difficulty, check the logic and accuracy of outputs, and revise their own work, while retaining responsibility for interpretation, evaluation, and academic judgement. Therefore, LCU captures the role of GenAI within students’ own academic activity, rather than merely indicating whether or how often students use GenAI (Chiu, 2024; Weng et al., 2024). To examine why this pattern may be academically relevant, the study considers self-regulated learning (SRL) and academic self-efficacy (ASE) as psychological resources, and academic procrastination (PRO) and learning engagement (ENG) as later indicators of academic functioning. This framing links GenAI use with students’ regulation, confidence, and academic action by focusing on how students organise, monitor, evaluate, and revise academic work with GenAI.

This study contributes to GenAI educational psychology in three ways. First, it shifts the analytic focus from whether students use GenAI, or how often they use it, to how they incorporate GenAI into academic task work. By conceptualising learning-centred use as a task-focused and non-substitutive practice, the study distinguishes regulatory use from answer-oriented reliance. Second, it specifies a process model in which LCU is linked to later academic functioning through SRL and ASE rather than through direct tool exposure. This helps explain why GenAI use may be academically meaningful for some students but not for others. Third, it strengthens the empirical basis for this process interpretation by using a baseline-adjusted three-wave panel design, module-separated SRL and ASE measures, Inverse probability weighting (IPW) sensitivity analyses, and auxiliary behavioural and rater indicators. Together, these contributions reposition GenAI use as a learner-regulated academic practice rather than a simple exposure variable, and they clarify why GenAI may be academically meaningful only when students retain responsibility for planning, monitoring, verification, and revision.

## 2 Literature review and theoretical hypotheses

This section develops theoretical logic linking LCU with later academic functioning. The argument proceeds in three steps. First, LCU is defined as a psychologically meaningful pattern that captures how students incorporate GenAI into academic tasks. Second, LCU is linked to SRL and ASE as two intermediate psychological resources. Third, SRL and ASE are connected to later PRO and ENG as complementary indicators of academic functioning.

### 2.1 Learning-centred use of GenAI in academic work as a psychologically meaningful use pattern

LCU can be situated within research on external assistance and the difference between tool-supported performance and learner development. In educational psychology, help seeking is considered adaptive when students identify difficulties, seek support strategically, and remain cognitively and motivationally engaged in the task (Newman, 2002). Work on intelligent tutoring systems reaches a similar conclusion: assistance has educational value when it sustains problem solving and metacognitive control rather than enabling learners to bypass the learning process (Aleven et al., 2006). This perspective is particularly relevant to GenAI because conversational systems provide immediate and fluent support that may either extend students’ thinking or substitute for it (Izquierdo-Condoy et al., 2025).

Salomon et al.’s (1991) distinction between effects with a technology and effects of a technology further clarifies this issue. GenAI may support task completion during use, but such support is educationally meaningful only when it contributes to more durable forms of reasoning, monitoring, and judgement. The risk is that fluent outputs can create an appearance of adequate task completion before the learner has evaluated the underlying reasoning (Murugesan and Cherukuri, 2023). Cognitive offloading research similarly cautions that external tools may reduce cognitive demand when learners delegate too much intellectual work to the tool (Risko and Gilbert, 2016). Recent GenAI research develops this concern by emphasising the need for critical evaluation, fact-checking, and human oversight (Kasneci et al., 2023), while also showing that GenAI activities may relate to phases of SRL and vary across learners (Chiu, 2024). Scaffolded use may preserve cognitive friction and learner agency, whereas unstructured use may encourage metacognitive disengagement and epistemic narrowing (Vendrell and Johnston, 2026).

On this basis, LCU is conceptualised in this study as an agency-preserving pattern of human–AI academic interaction. It concerns how epistemic labour is allocated between the learner and the system. In learning-centred use, students use GenAI to clarify, compare, check, and revise, while treating AI outputs as provisional materials for inspection, adaptation, or rejection. Responsibility for disciplinary standards, interpretation, and final academic judgement remains with the student (Flenady and Sparrow, 2025). This framing positions LCU as a task-level practice within the learner–tool–task relationship. It is more specific than broad technology acceptance because it concerns how students conduct academic work after GenAI becomes available. It is also distinct from SRL, which refers to the broader regulation of goals, strategies, monitoring, and effort.

This distinction is important for the psychological logic of the study. LCU is not treated as a general learner trait or as another name for self-regulation. It is a situated, tool-mediated form of strategic help seeking in which the learner remains responsible for defining the problem, judging the value of assistance, and transforming external support into self-owned academic action. In social-cognitive terms, LCU describes behaviour within a person–tool–task environment, whereas SRL describes the learner-level system through which goals, monitoring, strategy adjustment, and effort are organised across academic contexts. The model therefore tests whether a particular pattern of GenAI-supported task practice is associated with later regulation, rather than assuming that LCU and SRL are the same construct.

### 2.2 Learning-centred use and self-regulated learning

GenAI creates a distinctive setting because assistance is immediate, interactive, and open-ended. Such assistance can reduce uncertainty, but it may undermine LCU when students accept outputs before examining their relevance or quality (Sohail et al., 2025). SRL provides a useful perspective for understanding these challenges. SRL involves goal setting, planning, monitoring, strategy adjustment, and persistence under difficulty (Zimmerman, 2002; Pintrich, 2004; Panadero, 2017). In GenAI-supported academic work, LCU translates these processes into purposeful tool use, critical judgement of outputs, and revision guided by task goals. Recent research treats GenAI as a conditional support for SRL. This conditional view operates at two levels. First, it concerns design: GenAI-based activities can be connected to the phases of SRL when they are deliberately organised around learning purposes (Chiu, 2024). Second, it concerns the regulatory process: metacognitive support in GenAI environments can help students develop task strategies, strengthen self-evaluation, and reduce unexamined dependence on tool outputs (Xu et al., 2025). At a broader level, Xia et al. (2026) and Lan and Zhou (2025) reach a similar conclusion: GenAI may support forethought, performance, and reflection when students remain active agents in planning, monitoring, and evaluating their own learning.

This perspective clarifies why LCU is expected to relate to later SRL. LCU does not simply add another source of help to academic work. It requires students to decide when assistance is needed, what kind of response is useful, whether the response fits the task, and how the response should be transformed into their own academic action. These decisions keep regulatory responsibility with the learner. When students repeatedly use GenAI in this way, they practice the judgement and control needed to manage academic difficulty (Hou et al., 2025). Accordingly, stronger LCU at T1 is expected to be positively associated with SRL at T2. The expected link rests on students’ active governance of GenAI-mediated assistance, rather than on GenAI availability or task efficiency alone.

The proposed LCU-to-SRL link is also consistent with metacognitive accounts of learning. When students ask GenAI for clarification, compare alternative solutions, check claims, and revise their own work, they engage in the same classes of monitoring and control operations that SRL theories identify as central to adaptive learning. However, these operations are expressed through interaction with an external tool. This is why LCU is conceptualised as a task-level practice that can provide opportunities for regulatory activity, while SRL is conceptualised as the broader psychological mechanism that organises that activity over time.

### 2.3 Learning-centred use, self-regulated learning, and academic self-efficacy

ASE refers to students’ beliefs that they can successfully manage academic demands (Bandura, 1997; Honicke and Broadbent, 2016). In GenAI-supported learning, the key issue is whether such confidence is grounded in students’ own academic action. Recent evidence suggests that AI-assisted learning and GenAI literacy may be associated with stronger ASE, especially when students also show motivation, critical thinking, and active engagement with the learning process (Shao and Wang, 2026; Wang, 2026). However, this relationship requires caution. GenAI can make task completion faster and smoother, and this ease may produce inflated confidence when students rely on generated outputs without understanding or evaluating the underlying reasoning. Such reliance may strengthen false self-efficacy while weakening independent academic performance (Bae and Bozkurt, 2024).

LCU is relevant to ASE because it frames AI support as a source of controlled evidence. Drawing on Bandura’s (1997) view that efficacy beliefs are shaped by mastery experience, students gain efficacy-relevant information when they judge whether an AI response is useful, adapt it to task requirements, and recognise how their own decisions improve the academic product. In this sense, confidence is grounded in managed progress and extends beyond output fluency alone. SRL further explains how ASE may develop. Social cognitive theory suggests that regulated academic action can provide repeated mastery-relevant experiences through which efficacy beliefs may develop (Schunk, 1990; Schunk and Swartz, 1993). In GenAI-supported work, students who can govern the interaction, evaluate interim responses, and recover from difficulty through strategic control are more likely to interpret academic demands as manageable. Accordingly, SRL and ASE are treated as distinct but complementary mechanisms: SRL concerns the organisation and monitoring of academic action, whereas ASE concerns students’ judgement of capability. This paper therefore places SRL before ASE for theoretical reasons. In the present design, SRL and ASE were separated into T2a and T2b modules with a short interval, strengthening the theory-specified ordering while still requiring cautious interpretation because the study remains observational.

From a social-cognitive perspective, the quality of the evidence on which efficacy beliefs are built matters. If students experience smooth task completion only because GenAI supplies an answer, the resulting confidence may be weakly grounded. By contrast, when students use GenAI to test ideas, evaluate feedback, correct errors, and observe their own successful regulation of the task, the experience is more likely to provide mastery-relevant information. This is why ASE is positioned as a capability-judgement resource linked to, but distinct from, SRL. SRL concerns the organisation of action; ASE concerns students’ interpretation of whether they can manage similar demands in the future.

### 2.4 Self-regulated learning, academic self-efficacy, and later academic functioning

The present study examines academic PRO and ENG as two indicators of later academic functioning. PRO reflects delay in initiating or completing important academic work despite expected costs and is widely understood as a self-regulatory difficulty (Steel, 2007; Sirois and Pychyl, 2013). ENG reflects energy, concentration, and sustained investment in learning activity (Fredricks et al., 2004). Considering both outcomes is useful because reduced delay and increased engagement do not necessarily develop together. Some students may complete required work with limited involvement, whereas others may show interest while still struggling to regulate delay. Examining both outcomes avoids reducing academic functioning either to the absence of delay or the presence of visible activity.

The pairing of PRO and ENG also reflects a psychological distinction between avoidance and investment. Procrastination is not simply low engagement; it involves delay despite anticipated negative consequences and is often linked to failures of self-regulation and short-term mood repair. Engagement, in contrast, represents the positive mobilisation of energy, attention, and persistence toward academic activity. Examining both outcomes allows the study to test whether the same regulatory and efficacy-related resources are connected to reduced delay and increased investment, without assuming that these two forms of functioning are mirror images of each other.

As argued in Section 2.3, LCU is expected to be connected with SRL and ASE. These resources are also relevant to PRO and ENG. SRL concerns how students manage academic action under difficulty. Students who can clarify goals, monitor progress, adjust strategies, and persist after setbacks are better positioned to begin tasks, maintain effort, and recover when work becomes demanding (Seli, 2019). This expectation is consistent with higher education evidence linking SRL-related support with more adaptive ENG and learning functioning (Wolters et al., 2023). ASE adds a capability-related resource. Students who judge academic demands as manageable are more likely to sustain effort and remain engaged, which is consistent with evidence linking ASE with ENG and performance-related outcomes (Honicke and Broadbent, 2016; Fatimah et al., 2024).

Given that students use GenAI for academic activities in educational settings, this paper regards teachers’ guidance and support (TGS) as a contextual condition. Teachers may shape GenAI use through recommendations, task design, assessment criteria, and feedback practices (Fang and Cai, 2025). Such instructional environments are important because they help define the perceived meaning, legitimacy, and appropriate boundaries of AI use in academic work. However, students may still incorporate GenAI into their own study routines in different ways even under similar guidance conditions. Thus, TGS, GenAI-use frequency, and background characteristics are included as controls, allowing LCU to be examined while accounting for its instructional and behavioural context.

### 2.5 Hypotheses

The theoretical expectation is that LCU becomes academically relevant when it is connected with students’ SRL and ASE. In this paper, LCU may help students clarify, organise, check, and revise academic work, but later PRO and ENG depend more directly on students’ regulation of academic action and their beliefs about managing academic demands. This process-based expectation is consistent with a cautious interpretation of GenAI as a tool embedded in student activity rather than as an independent cause of academic functioning (Weston et al., 2025). Accordingly, the study advances the following hypotheses:

*H1*: T1 LCU affects T2a SRL positively.

*H2*: T1 LCU has a positive effect on T2b ASE.

*H3*: T2a SRL affects T2b ASE positively in the theoretically ordered SRL-to-ASE mechanism.

*H4a*: T2a SRL has a negative effect on T3 PRO.

*H4b*: T2b ASE affects T3 PRO negatively.

*H5a*: T2a SRL has a positive effect on T3 ENG.

*H5b*: T2b ASE affects positively T3 ENG.

*H6*: T2a SRL plays a mediating role between T1 LCU and T3 PRO, as well as between T1 LCU and T3 ENG, with T2b ASE functioning as a complementary capability-judgement mechanism and as part of the SRL-to-ASE pathway.

**Figure 1:**
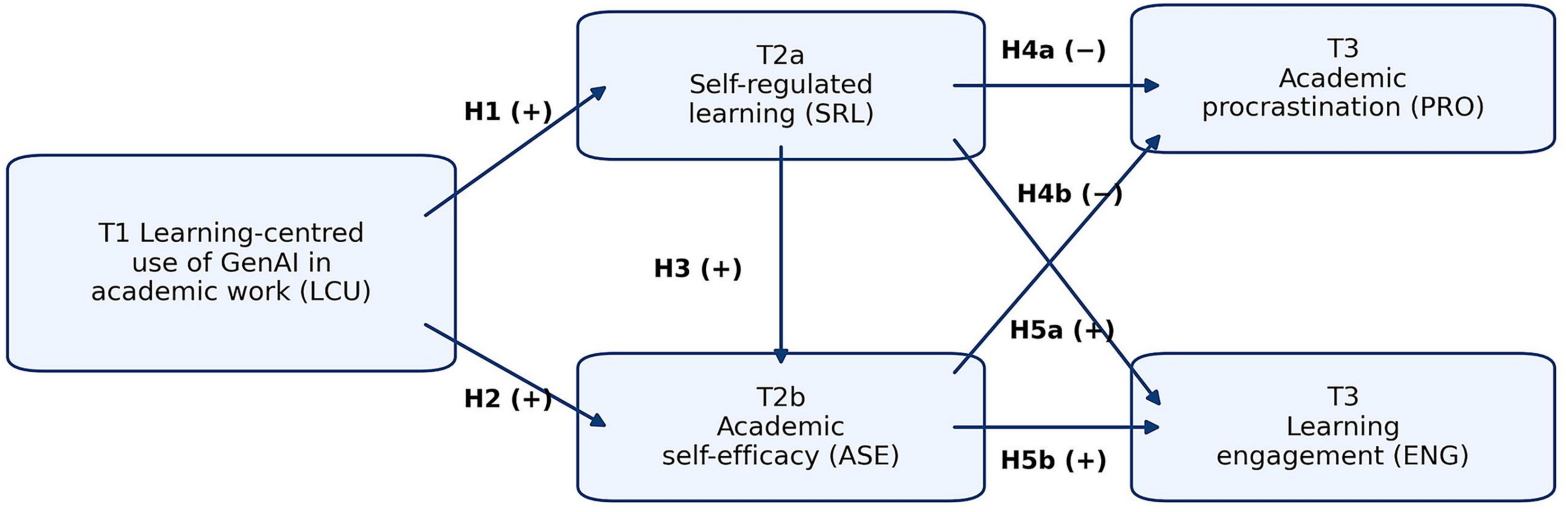
Conceptual model and hypothesised three-wave process structure. The model specifies LCU as the T1 GenAI use pattern, T2a SRL and T2b ASE as module-separated psychological mechanisms, and T3 PRO and ENG as functioning indicators. Baseline measures and covariates were controlled in the empirical model where appropriate.

Figure 1 summarises the conceptual model and the three-wave process structure used to organise the hypotheses.

## 3 Materials and methods

### 3.1 Data source and study design

The study used a de-identified three-wave online survey dataset collected from students enrolled in multiple higher education institutions in Sichuan, China. The research activity was conducted under the guidance and supervision of the Sichuan Construction Vocational Education Group and was approved as a minimal-risk student survey under Research Ethics Review Approval No. Sichuan Construction Vocational Education Group Research Ethics Review [2026] No. 052 (Supplementary material 1). Participants read an information sheet before answering the questionnaire and provided written or electronic informed consent. Responses were linked across waves using anonymised respondent IDs (Supplementary material 2).

The survey followed the process model developed in Sections 1 and 2. T1 measured demographic characteristics, GenAI-use profile, learning-centred use of GenAI in academic work (LCU), teacher guidance support (TGS), answer-oriented reliance (AOR), and baseline measures of self-regulated learning (SRL), academic self-efficacy (ASE), academic procrastination (PRO), and learning engagement (ENG). T2 was divided into two modules. T2a measured SRL, and T2b measured ASE. The two T2 modules were separated by 5–9 days, with a mean interval of approximately 7 days. T3 measured PRO and ENG. The intended interval between the main survey waves was approximately 4 weeks.

This design strengthens the three-wave structure in two ways. First, the inclusion of T1 SRL, ASE, PRO, and ENG allow the main models to control baseline psychological resources and baseline academic functioning. Second, separating SRL and ASE into T2a and T2b provides a clearer temporal basis for the theoretically specified SRL-to-ASE mechanism. The analyses therefore estimate baseline-adjusted, time-ordered pathway effects within an observational panel design.

### 3.2 Participants, panel retention, and data screening

The valid T1 panel frame contained 1,200 students after eligibility and data-quality screening. Screening criteria included eligibility for the student survey, successful anonymised follow-up matching, sufficient questionnaire completion, plausible response duration, absence of duplicate anonymised IDs, and passing wave-specific attention checks; Supplementary material 3 reports the valid panel flow used as the analytic denominator. T2 retained 984 valid respondents, and T3 retained 788 valid complete cases. The main analytic sample therefore included 788 students with valid T1, T2a, T2b, and T3 data. This reporting treats the 1,200-student T1 panel as the baseline sample and does not rely on the pre-screened response pool for substantive interpretation.

In the valid T1 sample, 50.67% of students identified as female, 47.83% as male, and 1.50% selected another/prefer-not-to-say option. Domestic students accounted for 97.00% of the baseline sample. Year levels were broadly distributed: Year 1, 26.42%; Year 2, 26.67%; Year 3, 24.92%; and Year 4 or above, 22.00%. The disciplinary profile was heterogeneous, with engineering and computer science as the largest group (28.75%), followed by management (16.67%), medicine and health (10.92%), natural sciences (10.00%), social sciences (9.50%), economics (7.42%), humanities (6.75%), arts (4.58%), agriculture (2.75%), and other fields (2.67%). Self-reported GPA bands were also distributed across four categories: <2.50 (15.25%), 2.50–2.99 (27.42%), 3.00–3.49 (36.25%), and ≥3.50 (21.08%).

**Figure 2:**
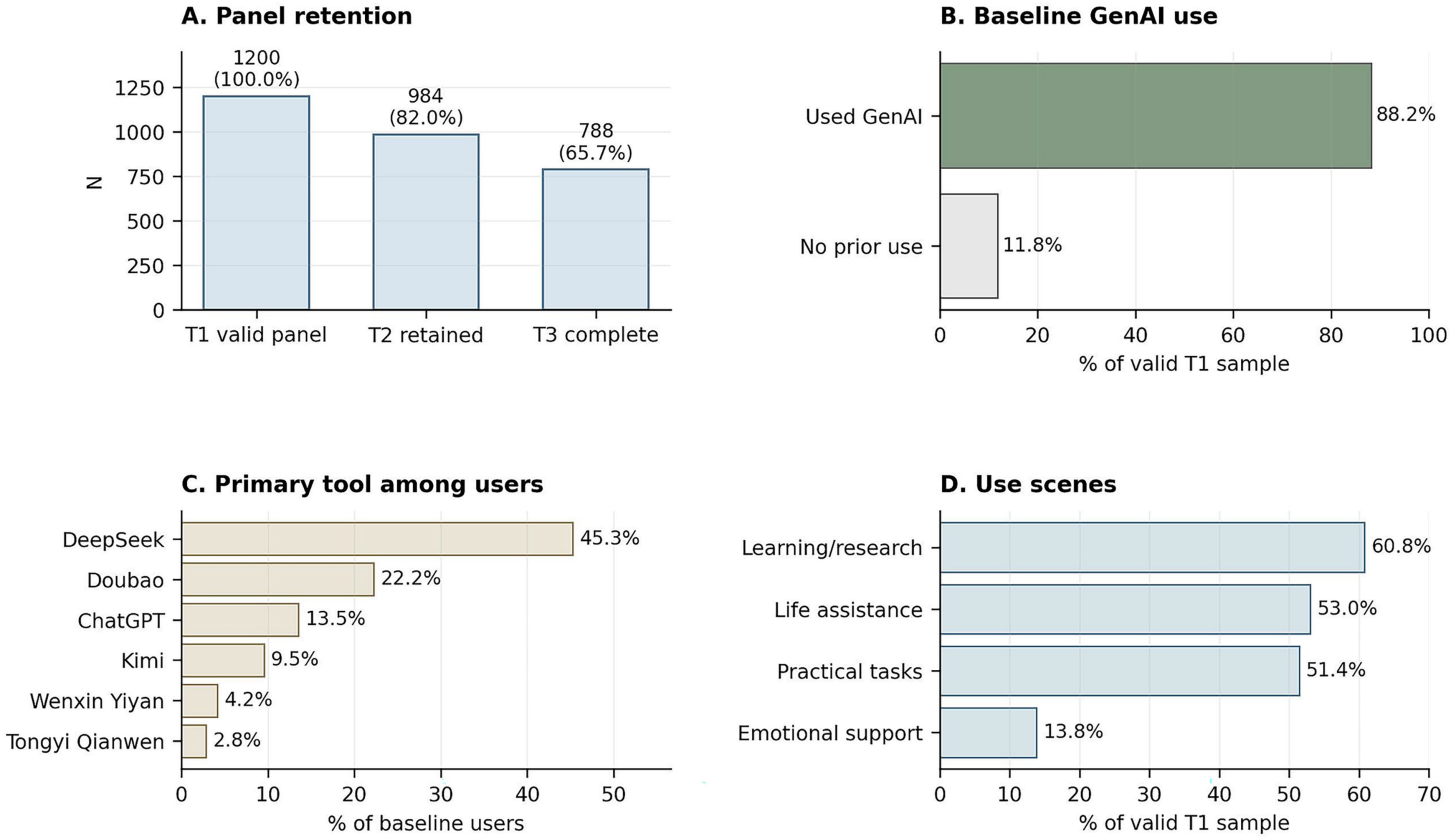
Panel retention and baseline GenAI-use ecology. Percentages for T2 and T3 are relative to the valid T1 baseline panel. Main-tool percentages are calculated among baseline GenAI users; use-scene percentages are calculated for the valid T1 sample and are not mutually exclusive.

Baseline GenAI use was common but not universal. In the T1 sample, 88.17% of students reported that they had used at least one GenAI tool. DeepSeek was the most frequently reported primary tool, followed by Doubao, ChatGPT, Kimi, Wenxin Yiyan, Tongyi Qianwen, and other tools. Learning or research was the most common use scene (60.75%), followed by life assistance (53.00%), practical tasks such as projects, presentations, code, or data analysis (51.42%), and emotional support (13.83%). These descriptive patterns indicate that the sample reflected a multi-tool and multi-scene GenAI ecology. Figure 2 summarises panel retention and the baseline GenAI-use ecology within the valid T1 panel frame.

Panel retention was examined before model estimation. Compared with students lost before T3, retained students reported higher baseline LCU (*M* = 3.189 vs. 2.855, standardised mean difference = 0.518), higher baseline SRL (*M* = 3.237 vs. 2.901, standardised mean difference = 0.545), higher baseline ENG (*M* = 3.258 vs. 2.941, standardised mean difference = 0.501), lower baseline PRO (*M* = 2.781 vs. 3.074, standardised mean difference = −0.464), and higher GPA band (*M* = 2.735 vs. 2.434, standardised mean difference = 0.306). The retention logit model also indicated that LCU, baseline SRL, baseline PRO, baseline ENG, baseline GenAI-user status, and GPA band were associated with retention. For this reason, complete-case models were reported as the primary analyses, and IPW models were estimated as a sensitivity check. Selective retention is treated as a limitation for generalisability.

### 3.3 Measures

The questionnaire was developed in Chinese for university students and organised by wave. All focal item sets used five-point response options, with higher scores indicating more of the corresponding measure. Composite scores were calculated as arithmetic means of retained items when sufficient item responses were available. Item-level missingness was low across the focal item sets. The measures were designed as brief, context-adapted indicators suitable for the present process model; further scale validation in independent samples remains necessary.

Learning-centred use of GenAI in academic work. LCU was measured at T1 with six items developed for the present GenAI context. The items asked whether students used GenAI to explain difficult concepts, organise ideas and steps before beginning work, reorganise learning materials, obtain hints or alternative approaches when stuck, inspect expression or logic before submitting an assignment, and treat GenAI as an auxiliary tool in academic tasks rather than as the final judge. The internal consistency of the six-item LCU measure was acceptable to strong (Cronbach’s *α* = 0.872, *M* = 3.074, SD = 0.655). A higher score indicated stronger learning-centred, task-focused, and non-substitutive use of GenAI in academic work.

Teacher guidance support. TGS was measured at T1 with four items asking whether teachers explained appropriate situations for AI use, reminded students to verify AI outputs, designed AI-plus-human assignments, and provided concrete feedback beyond simple prohibition. The scale showed acceptable internal consistency (*α* = 0.730, *M* = 2.910, SD = 0.684). TGS was included as a contextual covariate because instructional guidance may shape the perceived legitimacy, direction, and boundaries of students’ GenAI use.

Answer-oriented reliance. AOR was measured at T1 with four items describing answer-seeking or substitution-oriented reliance on GenAI. It was included as a contrast measure to distinguish LCU from patterns in which students use GenAI mainly to obtain answers, reduce their own effort, or accept generated output with limited evaluation. Conceptually, AOR represents a more substitutive allocation of epistemic responsibility to the tool, whereas LCU represents a learning-centred allocation in which students retain responsibility for checking, revision, and final judgement. The internal consistency was marginal to acceptable (*α* = 0.698, *M* = 2.569, SD = 0.658).

Baseline psychological resources and academic functioning. T1 baseline SRL, ASE, PRO, and ENG were measured to control for pre-existing individual differences. Baseline SRL was measured with six items (*α* = 0.815, *M* = 3.122, SD = 0.636), baseline ASE with five items (*α* = 0.760, *M* = 3.092, SD = 0.643), baseline PRO with six items (*α* = 0.791, *M* = 2.881, SD = 0.637), and baseline ENG with four items (*α* = 0.747, *M* = 3.149, SD = 0.664).

Self-regulated learning. SRL was measured at T2a with six items covering goal setting, advance planning, comprehension monitoring, strategy adjustment, pre-deadline progress checking, and persistence when tasks were difficult. The internal consistency was acceptable (*α* = 0.811, *M* = 3.169, SD = 0.636). T2a SRL was treated as the primary task-regulation mechanism.

Academic self-efficacy. ASE was measured at T2b with five items assessing confidence in mastering difficult course material, finding solutions to complex assignments, meeting course requirements under workload pressure, completing academic tasks independently, and achieving desired course performance when appropriate methods were used. The T2b ASE measure showed acceptable internal consistency (*α* = 0.752, *M* = 3.150, SD = 0.628). T2b ASE was treated as the capability-judgement mechanism following SRL in the theory-specified process model.

Academic procrastination and learning engagement. PRO was measured at T3 with six items capturing delayed task initiation, coursework postponement, waiting until deadlines were near, last-minute reviewing, attention diversion during difficult tasks, and difficulty completing academic work without external pressure. The internal consistency was acceptable (*α* = 0.796, *M* = 2.757, SD = 0.655). ENG was measured at T3 with four items assessing energy, focus, immersion, and willingness to invest sustained effort in current learning tasks (*α* = 0.740, *M* = 3.225, SD = 0.662).

Auxiliary behavioural and rater indicators. Several auxiliary indicators were collected for convergent evidence where consent and data availability permitted. At T2, 506 students consented to prompt-log use, and usable prompt-log records were available for 476 students. Verification prompt ratio was calculated as the proportion of academic GenAI prompts that involved checking, verification, revision, or evaluation rather than simple answer generation. Revision-trail quality was available for 385 students and was coded from anonymised revision-trail records using a rubric covering task clarification, output checking, revision, and independent judgement; two ratings were averaged when available, and the two rater scores were strongly correlated (*r* = 0.897). At T3, auxiliary indicators included late submission count, on-time submission rate, learning-management-system study sessions, and teacher engagement ratings where available. These indicators were not part of the primary path model and were used only to assess whether the self-report measures aligned with behaviourally adjacent evidence.

The full English reporting version of the questionnaire is provided in Supplementary material 2. Additional measurement diagnostics are reported in Supplementary Tables S1 and S2. Supplementary Table S1 summarises complete-item N, Cronbach’s alpha, loading ranges, composite reliability, and AVE. Supplementary Table S2 reports the item-level standardised loadings. Because several item sets were brief and adapted to the present GenAI context, these diagnostics are used to document measurement adequacy for the present process analyses rather than to claim full scale validation.

### 3.4 Data analysis strategy

The analysis proceeded in five stages. First, sample flow, descriptive statistics, panel retention, and internal consistency were examined using the valid T1 baseline panel as the starting point. The T1-to-T3 analytic sample was defined as students with valid T1 predictor data, valid T2a SRL, valid T2b ASE, and valid T3 PRO/ENG. Retention differences were examined through baseline mean comparisons and a logistic retention model. Stabilised IPW weights were then derived from the retention model and used in sensitivity analyses.

Second, zero-order correlations among focal measures were examined to check whether the bivariate pattern was consistent with the theoretical model. Because the study used brief context-adapted measures, reliability and measurement diagnostics were examined before the path models were interpreted. Supplementary material 2 reports the English item wording, and Supplementary Tables S1 and S2 report complete-item N, standardised item loadings, Cronbach’s alpha, composite reliability, and AVE for the focal measures. Discriminant validity was examined using Fornell–Larcker comparisons (Fornell and Larcker, 1981) and HTMT ratios (Henseler et al., 2015). These diagnostics were interpreted together with item content and the time-separated design, rather than through a single coefficient alone. Because the focal constructs were primarily self-reported, common-method risk was addressed through temporal separation, baseline adjustment, and auxiliary behavioural/rater convergence. These design and diagnostic choices reduce, but do not remove, the possibility of common-method influence (Podsakoff et al., 2003).

Third, the primary model was estimated as a baseline-adjusted composite-score path model using standardised scale means. The key predictor was T1 LCU. The mechanism variables were T2a SRL and T2b ASE. The outcomes were T3 PRO and T3 ENG. The model controlled for baseline SRL when predicting T2a SRL, baseline ASE when predicting T2b ASE, baseline PRO when predicting T3 PRO, and baseline ENG when predicting T3 ENG. Additional covariates included gender, student type, year level, discipline, GPA band, baseline GenAI-use frequency, and TGS. HC3 heteroskedasticity-consistent standard errors were used.

Fourth, indirect effects from LCU to PRO and ENG through SRL, ASE, and the SRL-to-ASE pathway were estimated from the baseline-adjusted path coefficients. Monte Carlo confidence intervals were used to evaluate the uncertainty around each indirect association. An indirect association was treated as statistically supported when the 95% confidence interval did not include zero.

Fifth, robustness and convergent-evidence checks were conducted. IPW models were estimated to assess whether observed retention differences altered the main pattern. An additional AOR-adjusted model was estimated to examine whether the focal LCU pathways were distinct from answer-oriented reliance on GenAI. The convergent-evidence analyses tested whether LCU correlated with verification prompt ratio and revision-trail quality, whether PRO correlated with late submission count and on-time submission rate, and whether ENG correlated with Learning Management System (LMS) study sessions and teacher engagement ratings. These checks were used to evaluate specification stability and measurement convergence, not to establish causal effects.

## 4 Results

This section presents the empirical results as standardised associations within a baseline-adjusted three-wave panel model. The results should be interpreted as prospective, time-ordered associations rather than as causal estimates. We first report sample flow, preliminary correlations, and retention diagnostics; we then present reliability evidence, the baseline-adjusted path model, indirect effects, IPW sensitivity analyses, and auxiliary behavioural and rater evidence.

### 4.1 Preliminary analyses, sample flow, and retention diagnostics

The final complete-case sample contained 788 students drawn from the valid T1 baseline panel of 1,200. T2 retained 984 valid respondents, and T3 retained 788 valid complete cases. The T1-to-T3 retention rate was 65.7%.

The zero-order correlations were consistent with the theoretical model. LCU was positively correlated with later SRL (*r* = 0.431, *p* < 0.01), ASE (*r* = 0.309, *p* < 0.01), and ENG (*r* = 0.270, *p* < 0.01), and negatively correlated with PRO (*r* = −0.215, *p* < 0.01). LCU was only weakly related to AOR (*r* = −0.029, *p* = 0.416), supporting the distinction between learning-centred and answer-oriented use patterns. AOR showed the opposite descriptive tendency, including weaker negative relations with SRL and ENG and a small positive relation with PRO. SRL and ASE were positively correlated (*r* = 0.375, *p* < 0.01), and both were associated with lower PRO and higher ENG.

Retention diagnostics showed that retained students differed from students lost before T3 on several baseline measures. Retained students reported higher LCU, baseline SRL, baseline ASE, baseline ENG, baseline GenAI-user status, AI-use frequency, and GPA band, and lower baseline PRO. In the logistic retention model, LCU (odds ratio = 1.673, *p* < 0.001), baseline SRL (odds ratio = 1.587, *p* < 0.001), baseline PRO (odds ratio = 0.706, *p* = 0.005), baseline ENG (odds ratio = 1.403, *p* = 0.004), GenAI-user status (odds ratio = 2.592, *p* = 0.001), and GPA band (odds ratio = 1.294, *p* < 0.001) were associated with retention. These results indicate selective retention. Complete-case models are reported as the main analyses, with IPW models used to assess whether the main results were sensitive to observed retention differences.

These retention patterns indicate that attrition was not random. The complete-case sample probably over-represents students who were already more engaged, more self-regulated, more familiar with GenAI, and academically stronger at baseline. This selection pattern may narrow the range of academic functioning observed at T3 and may make the estimates less informative for students with weaker regulatory profiles, lower engagement, lower GPA, or no prior GenAI use. The IPW analyses were therefore used to examine whether the focal pathway estimates were sensitive to observed retention differences. These weights cannot, however, remove bias from unobserved reasons for attrition.

### 4.2 Reliability and measurement evidence

Internal consistency estimates and descriptive statistics for the focal measures are reported in Table 1. Internal consistency was acceptable for all focal item sets. LCU showed strong reliability (*α* = 0.872). TGS showed acceptable reliability (*α* = 0.730), and AOR showed marginal to acceptable reliability (*α* = 0.698). Baseline SRL, ASE, PRO, and ENG also showed acceptable reliability (*α* = 0.815, 0.760, 0.791, and 0.747, respectively). At the follow-up waves, SRL at T2a (*α* = 0.811), ASE at T2b (*α* = 0.752), PRO at T3 (*α* = 0.796), and ENG at T3 (*α* = 0.740) showed acceptable internal consistency.

**Table 1:** Internal consistency and descriptive statistics for focal measures.

Measure	Wave/module	Items	*α*	*M*	SD	*N*
LCU	T1	6	0.872	3.074	0.655	1,200
TGS	T1	4	0.730	2.910	0.684	1,200
AOR	T1	4	0.698	2.569	0.658	1,200
SRL baseline	T1	6	0.815	3.122	0.636	1,200
ASE baseline	T1	5	0.760	3.092	0.643	1,200
PRO baseline	T1	6	0.791	2.881	0.637	1,200
ENG baseline	T1	4	0.747	3.149	0.664	1,200
SRL	T2a	6	0.811	3.169	0.636	984
ASE	T2b	5	0.752	3.150	0.628	984
PRO	T3	6	0.796	2.757	0.655	788
ENG	T3	4	0.740	3.225	0.662	788

LCU, learning-centred use of GenAI in academic work; TGS, teacher guidance support; AOR, answer-oriented reliance; SRL, self-regulated learning; ASE, academic self-efficacy; PRO, academic procrastination; ENG, learning engagement.

Overall, the reliability evidence was adequate for the present process analyses, but the measurement evidence was not uniformly strong. LCU had satisfactory internal consistency, composite reliability, and AVE. For several other brief, context-adapted scales, internal consistency and composite reliability were generally acceptable, and the item loadings were within a usable range, but AVE values were below the conventional 0.50 threshold. This indicates that convergent validity was limited for some measures. The HTMT results nevertheless supported empirical separation among the focal constructs: the largest HTMT ratio was 0.616 for SRL–ENG, and the LCU–SRL ratio was 0.518. We therefore interpret the measures as brief operational indicators rather than fully validated scales. Supplementary Tables S1 and S2 report the measurement diagnostics, and Supplementary Table S4 reports discriminant-validity diagnostics for the primary focal constructs.

### 4.3 Baseline-adjusted path model

The baseline-adjusted composite-score path model provided the primary test of the hypotheses. Table 2 reports the focal paths, corresponding baseline predictors, and key model controls. Full dummy-coded demographic and discipline covariates are reported in Supplementary Table S3.

**Table 2:** Baseline-adjusted path coefficients, including baseline predictors and key model controls.

Outcome	Predictor	*β*	SE	*t*	*p*	95% CI	Adjusted *R*^2^
SRL T2a	LCU T1	0.345	0.034	10.058	<0.001	[0.277, 0.412]	0.266
SRL T2a	SRL T1 baseline	0.257	0.033	7.865	<0.001	[0.193, 0.321]	
SRL T2a	GenAI-use frequency T1	−0.049	0.036	−1.375	0.169	[−0.119, 0.021]	
SRL T2a	TGS T1	0.145	0.031	4.631	<0.001	[0.083, 0.206]	
SRL T2a	GPA band	0.024	0.032	0.736	0.462	[−0.039, 0.087]	
ASE T2b	LCU T1	0.118	0.039	3.048	0.002	[0.042, 0.193]	0.253
ASE T2b	SRL T2a	0.244	0.036	6.834	<0.001	[0.174, 0.314]	
ASE T2b	ASE T1 baseline	0.262	0.033	7.971	<0.001	[0.198, 0.327]	
ASE T2b	GenAI-use frequency T1	0.044	0.034	1.287	0.198	[−0.023, 0.111]	
ASE T2b	TGS T1	0.119	0.036	3.353	<0.001	[0.050, 0.189]	
ASE T2b	GPA band	0.085	0.033	2.576	0.010	[0.020, 0.150]	
PRO T3	LCU T1	−0.006	0.037	−0.165	0.869	[−0.079, 0.067]	0.317
PRO T3	SRL T2a	−0.265	0.037	−7.163	<0.001	[−0.337, −0.192]	
PRO T3	ASE T2b	−0.207	0.033	−6.190	<0.001	[−0.273, −0.141]	
PRO T3	PRO T1 baseline	0.333	0.030	11.124	<0.001	[0.275, 0.392]	
PRO T3	GenAI-use frequency T1	0.007	0.032	0.203	0.839	[−0.057, 0.070]	
PRO T3	TGS T1	0.018	0.032	0.574	0.566	[−0.044, 0.080]	
PRO T3	GPA band	−0.043	0.031	−1.402	0.161	[−0.103, 0.017]	
ENG T3	LCU T1	0.009	0.038	0.229	0.819	[−0.066, 0.083]	0.310
ENG T3	SRL T2a	0.351	0.036	9.826	<0.001	[0.281, 0.421]	
ENG T3	ASE T2b	0.103	0.035	2.967	0.003	[0.035, 0.171]	
ENG T3	ENG T1 baseline	0.249	0.031	8.089	<0.001	[0.188, 0.309]	
ENG T3	GenAI-use frequency T1	0.019	0.034	0.541	0.588	[−0.049, 0.086]	
ENG T3	TGS T1	0.073	0.033	2.210	0.027	[0.008, 0.138]	
ENG T3	GPA band	0.068	0.033	2.066	0.039	[0.003, 0.132]	

Models used standardised composite scores and HC3 standard errors. GPA band was treated as an ordinal covariate. Gender, student type, year level, and discipline were included as dummy-coded covariates; their coefficients are reported in Supplementary Table S3 to keep the main table readable. The reference groups were female, domestic student, Year 1, and engineering/computer science. Adjusted *R*^2^ values were 0.266 for SRL T2a, 0.253 for ASE T2b, 0.317 for PRO T3, and 0.310 for ENG T3.

For H1, T1 LCU showed a positive path effect on T2a SRL after baseline SRL and covariates were controlled (*β* = 0.345, SE = 0.034, *p* < 0.001, 95% CI [0.277, 0.412]). Baseline SRL also predicted T2a SRL (*β* = 0.257, *p* < 0.001). These results support H1 and indicate that LCU predicted later SRL above students’ baseline self-regulation.

For H2, T1 LCU showed a positive path effect on T2b ASE after baseline ASE, T2a SRL, and covariates were controlled (*β* = 0.118, SE = 0.039, *p* = 0.002, 95% CI [0.042, 0.193]). This supports H2, although the LCU-to-ASE path was smaller than the LCU-to-SRL path.

For H3, T2a SRL showed a positive path effect on T2b ASE (*β* = 0.244, SE = 0.036, *p* < 0.001, 95% CI [0.174, 0.314]). Since SRL and ASE were measured in separate T2 modules with a 5–9 day interval, this result provides temporally separated evidence consistent with the theory-specified SRL-to-ASE mechanism. However, the short interval means that the path should not be read as evidence of long-term developmental change in efficacy beliefs. Baseline ASE remained associated with T2b ASE (*β* = 0.262, *p* < 0.001).

For the outcome models, T2a SRL and T2b ASE showed path effects on both T3 academic functioning indicators after baseline outcomes were controlled. For H4a and H4b, SRL negatively predicted T3 PRO (*β* = −0.265, SE = 0.037, *p* < 0.001, 95% CI [−0.337, −0.192]), and ASE also negatively predicted T3 PRO (*β* = −0.207, SE = 0.033, *p* < 0.001, 95% CI [−0.273, −0.141]). Baseline PRO remained a strong predictor of T3 PRO (*β* = 0.333, *p* < 0.001). These results support H4a and H4b.

For H5a and H5b, SRL positively predicted T3 ENG (*β* = 0.351, SE = 0.036, *p* < 0.001, 95% CI [0.281, 0.421]), and ASE also positively predicted T3 ENG (*β* = 0.103, SE = 0.035, *p* = 0.003, 95% CI [0.035, 0.171]). Baseline ENG remained associated with T3 ENG (*β* = 0.249, *p* < 0.001). These results support H5a and H5b.

Direct LCU-to-outcome path effects were not statistically supported once the mechanisms and baseline outcomes were included. The LCU-to-PRO path was close to zero (*β* = −0.006, *p* = 0.869), and the LCU-to-ENG path was also non-significant (*β* = 0.009, *p* = 0.819). This result is substantively important: the academic influence of LCU operated mainly through SRL and ASE, rather than through direct links from LCU to PRO or ENG.

**Figure 3:**
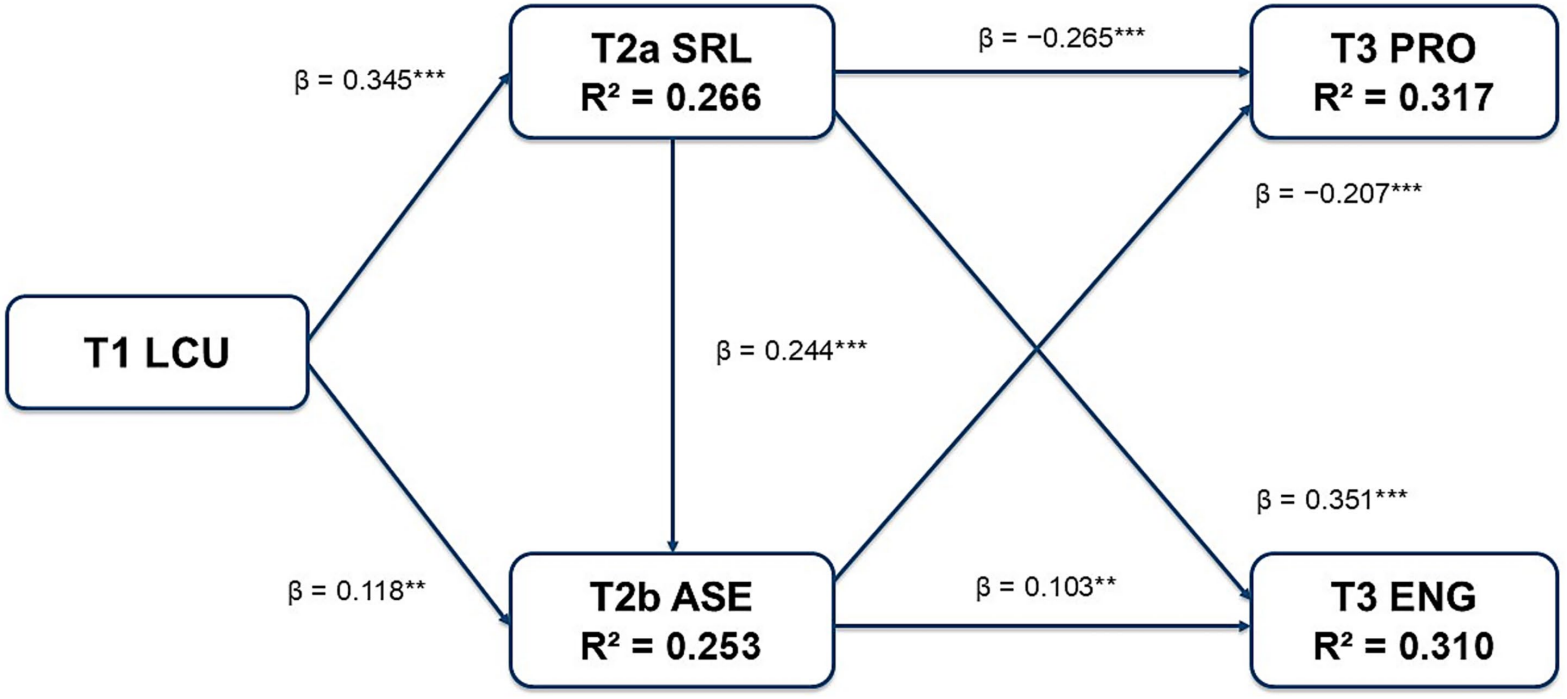
Core baseline-adjusted path model. Coefficients are rounded standardised path estimates for the focal mechanisms. The corresponding T1 baseline measure was controlled in each equation, together with gender, student type, year level, discipline, GPA band, baseline GenAI-use frequency, and TGS. Baseline predictors and key controls are reported in Table 2; full dummy-coded covariate coefficients are reported in Supplementary Table S3. Direct LCU-to-outcome paths were estimated but are not displayed because they were not statistically significant. Values in boxes are adjusted *R*^2^ values. ^**^p < 0.01, ^***^p < 0.001.

Because AOR was included to distinguish learning-centred from answer-oriented GenAI use, the baseline-adjusted model was also re-estimated with AOR entered as an additional T1 covariate. The focal LCU-to-SRL path remained statistically supported (*β* = 0.347, *p* < 0.001), as did the LCU-to-ASE path (*β* = 0.119, *p* = 0.002). AOR itself was not statistically significant in the SRL (*β* = 0.024, *p* = 0.486), ASE (*β* = 0.013, *p* = 0.693), PRO (*β* = 0.023, *p* = 0.455), or ENG (*β* = −0.042, *p* = 0.200) equations. This sensitivity check indicates that the main LCU mechanism was not reducible to answer-oriented reliance on GenAI. Figure 3 visualises the focal baseline-adjusted mechanism paths.

### 4.4 Indirect effects

H6 was evaluated using indirect effects calculated from the baseline-adjusted path coefficients. All theory-specified indirect effects were supported by confidence intervals excluding zero.

For PRO, LCU had a negative indirect association through SRL (standardised association = −0.091, 95% CI [−0.123, −0.062]), through ASE (standardised association = −0.024, 95% CI [−0.044, −0.008]), and through the SRL-to-ASE pathway (standardised association = −0.017, 95% CI [−0.027, −0.010]). For ENG, LCU had a positive indirect association through SRL (standardised association = 0.121, 95% CI [0.088, 0.156]), through ASE (standardised association = 0.012, 95% CI [0.003, 0.025]), and through the SRL-to-ASE pathway (standardised association = 0.009, 95% CI [0.003, 0.016]).

**Figure 4:**
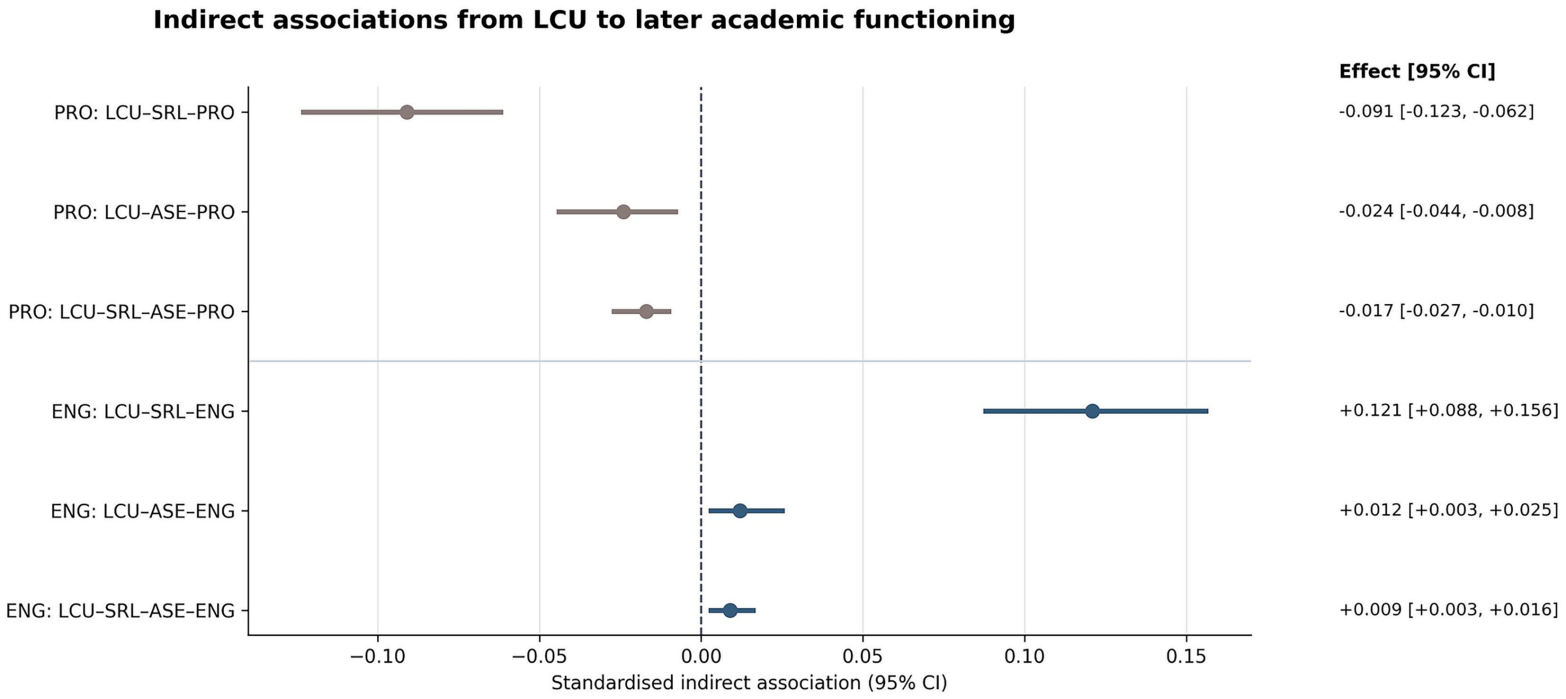
Indirect effects from LCU to later academic functioning. Points indicate standardised indirect effects; horizontal bars indicate 95% confidence intervals estimated from the baseline-adjusted path model.

The indirect effects are summarised in Table 3 and displayed graphically in Figure 4. The magnitude pattern indicates that SRL carried the largest specific indirect association for both outcomes. ASE also contributed, but its specific indirect effects were smaller. The serial SRL-to-ASE pathway was statistically supported for both PRO and ENG, consistent with the theory that regulated academic action can be linked to later capability judgement.

**Table 3:** Indirect effects from LCU to later academic functioning.

Outcome	Indirect path	Standardised association	95% CI	Supported
PRO	LCU → SRL → PRO	−0.091	[−0.123, −0.062]	Yes
PRO	LCU → ASE → PRO	−0.024	[−0.044, −0.008]	Yes
PRO	LCU → SRL → ASE → PRO	−0.017	[−0.027, −0.010]	Yes
ENG	LCU → SRL → ENG	0.121	[0.088, 0.156]	Yes
ENG	LCU → ASE → ENG	0.012	[0.003, 0.025]	Yes
ENG	LCU → SRL → ASE → ENG	0.009	[0.003, 0.016]	Yes

Confidence intervals were estimated using Monte Carlo simulation based on the baseline-adjusted path coefficients. Effects are interpreted as statistically supported when the 95% CI excludes zero.

### 4.5 IPW sensitivity analyses

IPW sensitivity models were estimated because retention was selective. Stabilised weights were derived from the retention model and inspected before estimation. To reduce the influence of a small number of cases with very low predicted retention probabilities, the final stabilised weights were capped at 2.772. In the retained analytic sample, the final weights had a mean of 0.999, a standard deviation of 0.349, a minimum of 0.677, a 99th percentile of 2.757, and a maximum of 2.772. The weighted models were then used to examine whether the focal pathway estimates were sensitive to observed retention differences. They produced the same substantive pattern as the complete-case baseline-adjusted models.

The IPW sensitivity path coefficients are reported in Table 4. In the IPW model, LCU retained positive path effects on T2a SRL (*β* = 0.337, SE = 0.037, *p* < 0.001, 95% CI [0.264, 0.409]) and T2b ASE (*β* = 0.127, SE = 0.041, *p* = 0.002, 95% CI [0.047, 0.208]). SRL retained a positive path effect on ASE (*β* = 0.242, *p* < 0.001). In the PRO equation, SRL (*β* = −0.278, *p* < 0.001), ASE (*β* = −0.200, *p* < 0.001), and baseline PRO (*β* = 0.329, *p* < 0.001) retained the expected pattern, while the direct LCU-to-PRO path remained non-significant (*β* = 0.014, *p* = 0.730). In the ENG equation, SRL (*β* = 0.360, *p* < 0.001), ASE (*β* = 0.104, *p* = 0.004), and baseline ENG (*β* = 0.260, *p* < 0.001) retained the expected pattern, while the direct LCU-to-ENG path remained non-significant (*β* = −0.011, *p* = 0.778).

**Table 4:** IPW sensitivity path coefficients.

Outcome	Predictor	*β*	SE	*p*	95% CI
SRL T2a	LCU T1	0.337	0.037	<0.001	[0.264, 0.409]
SRL T2a	SRL T1 baseline	0.257	0.034	<0.001	[0.189, 0.324]
ASE T2b	LCU T1	0.127	0.041	0.002	[0.047, 0.208]
ASE T2b	SRL T2a	0.242	0.037	<0.001	[0.170, 0.314]
ASE T2b	ASE T1 baseline	0.273	0.034	<0.001	[0.206, 0.340]
PRO T3	LCU T1	0.014	0.040	0.730	[−0.064, 0.092]
PRO T3	SRL T2a	−0.278	0.039	<0.001	[−0.354, −0.203]
PRO T3	ASE T2b	−0.200	0.035	<0.001	[−0.268, −0.132]
PRO T3	PRO T1 baseline	0.329	0.032	<0.001	[0.265, 0.392]
ENG T3	LCU T1	−0.011	0.040	0.778	[−0.091, 0.068]
ENG T3	SRL T2a	0.360	0.039	<0.001	[0.284, 0.436]
ENG T3	ASE T2b	0.104	0.036	0.004	[0.033, 0.175]
ENG T3	ENG T1 baseline	0.260	0.033	<0.001	[0.196, 0.324]

These results indicate that the main process pattern was not dependent on the unweighted complete-case specification. Selective retention remains relevant for generalisability, but the IPW models suggest that the observed retention differences did not overturn the focal LCU–SRL/ASE–functioning pattern.

### 4.6 Auxiliary behavioural and rater evidence

**Figure 5:**
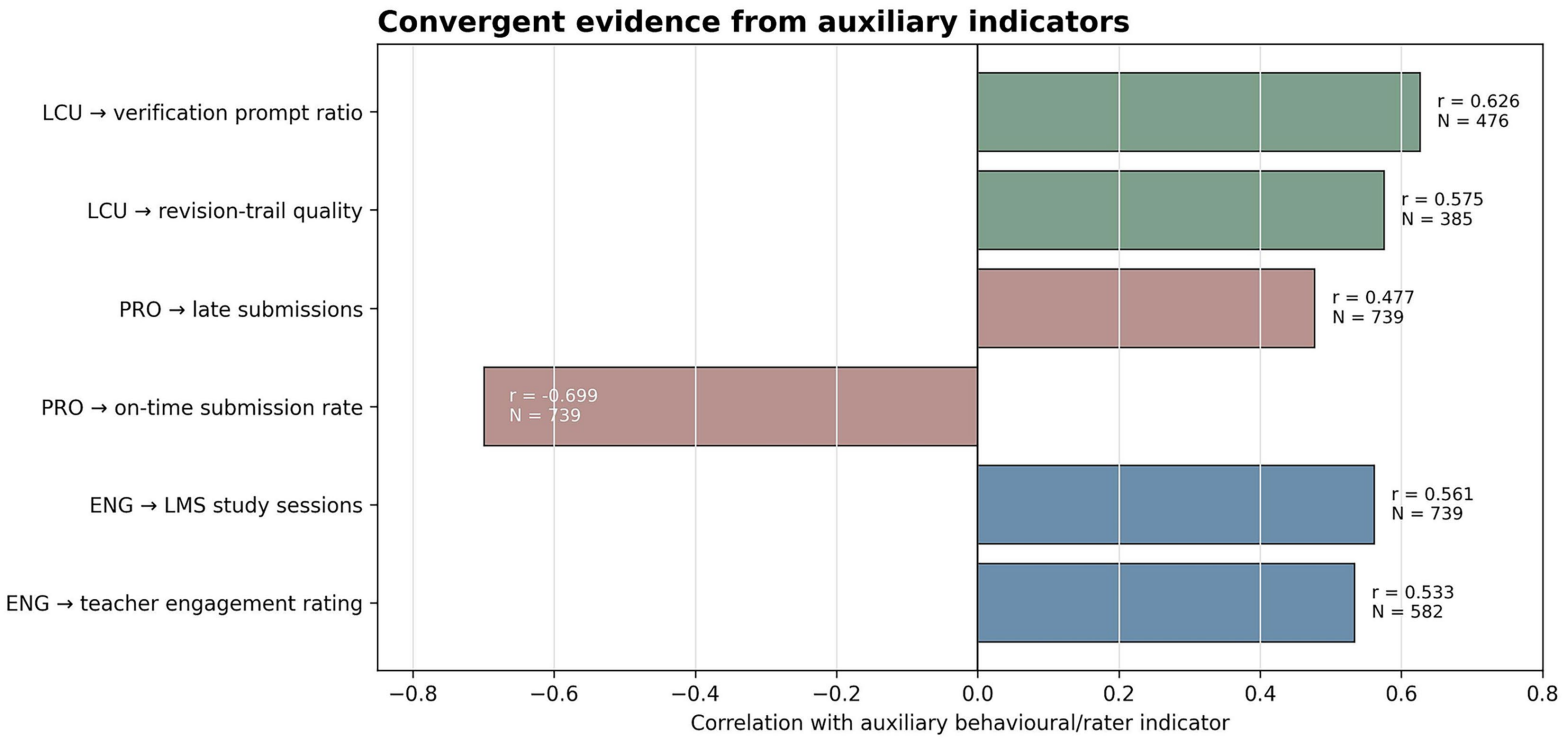
Convergent evidence from auxiliary behavioural and rater indicators. Correlations are pairwise correlations using available subsamples. Verification prompt ratio refers to the proportion of academic GenAI prompts involving checking, verification, revision, or evaluation. Revision-trail quality was coded from anonymised revision records using a rubric for task clarification, output checking, revision, and independent judgement. Auxiliary indicators were used as convergent evidence, not as replacements for the primary self-report measures.

Auxiliary behavioural and rater indicators provided convergent evidence for the self-report constructs, as summarised in Table 5 and Figure 5. LCU was strongly associated with verification prompt ratio (*r* = 0.626, pairwise *N* = 476) and revision-trail quality (*r* = 0.575, pairwise *N* = 385). Because these indicators were available only for consenting or data-available subsamples, they are not interpreted as additional primary outcomes. Rather, they show that students who reported higher LCU were also more likely to display checking, verification, and revision behaviours in GenAI-supported academic work.

**Table 5:** Auxiliary behavioural and rater correlations.

Self-report or theory variable	Auxiliary indicator	*N*	*r*	Interpretation
LCU T1	Verification prompt ratio T2	476	0.626	Higher LCU aligned with verification/checking behaviour
LCU T1	Revision-trail quality T2	385	0.575	Higher LCU aligned with stronger human–AI revision quality
PRO T3	Late submission count T3	739	0.477	Higher PRO aligned with more late submissions
PRO T3	On-time submission rate T3	739	−0.699	Higher PRO aligned with lower on-time submission rate
ENG T3	LMS study sessions T3	739	0.561	Higher ENG aligned with more study-platform activity
ENG T3	Teacher engagement rating T3	582	0.533	Higher ENG aligned with external engagement ratings

The outcome indicators also showed expected convergence with auxiliary behavioural measures. PRO was positively correlated with late submission count (*r* = 0.477, pairwise *N* = 739) and negatively correlated with on-time submission rate (*r* = −0.699, pairwise *N* = 739). ENG was positively correlated with LMS study sessions (*r* = 0.561, pairwise *N* = 739) and teacher engagement rating (*r* = 0.533, pairwise *N* = 582). These correlations do not replace the self-report measures, but they reduce the concern that the results are only artefacts of students’ self-descriptions.

### 4.7 Summary of hypothesis tests

The baseline-adjusted model supported H1–H6. LCU was associated with later SRL and ASE after baseline SRL and ASE were controlled. SRL was associated with later ASE after baseline ASE was controlled. SRL and ASE were associated with later PRO and ENG after baseline PRO and ENG were controlled. The direct paths from LCU to PRO and ENG were not significant in either the unweighted or IPW models. These results support a process-based interpretation: LCU was linked to later academic functioning through regulatory and efficacy-related resources.

## 5 Discussion

### 5.1 Main findings

This study examined whether learning-centred use of GenAI in academic work affected later academic functioning through SRL and ASE. The findings support the proposed time-ordered process model. T1 LCU showed positive path effects on T2a SRL and T2b ASE after baseline psychological resources and covariates were controlled. T2a SRL also showed a positive path effect on T2b ASE after baseline ASE was controlled. In turn, SRL and ASE predicted T3 PRO and ENG after baseline outcomes were controlled. The indirect effects from LCU to PRO and ENG through SRL, ASE, and the SRL-to-ASE sequence were statistically supported.

The direct paths from LCU to the T3 outcomes were not statistically significant after the mechanisms and baseline outcomes were included. This is central to the interpretation. The results do not show that GenAI use directly changed students’ academic functioning. They suggest that LCU was academically relevant when it was connected with students’ own regulation of academic action and their capability judgements. This interpretation is consistent with the article’s theoretical focus on GenAI as a tool embedded in learner activity.

The strongest specific indirect effects ran through SRL. This pattern indicates that LCU was most closely related to task-regulation activity: clarifying what the task requires, organising starting points, checking progress, using hints after difficulty, and revising work. ASE also contributed to the model, but its specific indirect effects were smaller. This supports the view that efficacy beliefs are relevant, while the more immediate mechanism lies in how students regulate academic work.

### 5.2 Contribution to GenAI educational psychology

The findings contribute to GenAI educational psychology by shifting attention from general use to the quality of academic use. Many studies measure GenAI use through adoption, frequency, perceived usefulness, attitude, or satisfaction. These indicators help describe technology acceptance, but they cannot distinguish students who use GenAI to clarify, check, and revise from students who use it to obtain answers with little evaluation. The present findings support this distinction in two ways: LCU remained associated with SRL and ASE after baseline GenAI-use frequency, TGS, baseline psychological resources, and baseline academic functioning were controlled; and the additional AOR sensitivity model showed that the LCU mechanism was not reducible to answer-oriented reliance.

Against this background, the present findings add a process-level perspective to international policy and adoption research on GenAI in higher education. Whereas policy-oriented and TAM/UTAUT-based studies explain institutional guidance and students’ adoption or continued use, LCU asks what students do with GenAI in academic tasks after access has become available (Wang et al., 2024; Jin et al., 2025; Sergeeva et al., 2025; Sousa and Cardoso, 2025). This keeps the contribution focused on verification, revision, and learner judgement rather than on acceptance or frequency alone.

The study also contributes by using a more time-ordered design than a single-wave survey model. T1 measured LCU and baseline measures, T2a measured SRL, T2b measured ASE, and T3 measured PRO and ENG. The separation between SRL and ASE provides temporally separated evidence consistent with the theory-specified SRL-to-ASE link. Meanwhile, the study remains observational, and the T2a–T2b interval was short; the findings should therefore be described as time-ordered pathway effects, rather than as experimental causality or long-term efficacy development.

A further contribution lies in the auxiliary behavioural and rater evidence. Higher LCU was associated with verification prompt ratio and revision-trail quality. Higher PRO was associated with more late submissions and lower on-time submission rate. Higher ENG was associated with more LMS study sessions and higher teacher engagement ratings. Because these indicators were available for subsamples, they do not replace experimental, log-based, or fully observed behavioural designs. They nevertheless provide useful convergent evidence that the focal self-report measures showed alignment with behaviourally adjacent indicators.

### 5.3 SRL and ASE as intermediate psychological resources

SRL appears to be the primary mechanism in the present model. The LCU-to-SRL path was stronger than the LCU-to-ASE path, and the SRL-specific indirect paths were the largest for both PRO and ENG. This suggests that LCU has academic relevance when students use GenAI in ways that keep planning, monitoring, strategy adjustment, and checking under their own responsibility.

This pattern also clarifies the boundary between LCU and SRL. LCU describes a way of engaging with an external tool during academic tasks; SRL describes the broader psychological system that organises goals, monitoring, strategy adjustment, and persistence. Their association is theoretically expected, but the constructs are not interchangeable. The results suggest that students’ tool-mediated practices can be linked with later learner-level regulation, rather than showing that the two constructs are identical.

ASE added a complementary mechanism. The LCU-to-ASE path was smaller than the LCU-to-SRL path, but it remained statistically supported after baseline ASE and SRL were included. The SRL-to-ASE path was also supported. This pattern is consistent with the theory that regulated academic action can provide mastery-relevant experiences from which efficacy beliefs may develop. In practical terms, students may feel more capable when they use GenAI to clarify task demands, test alternatives, verify outputs, and revise their own work while still making the final judgement themselves.

The outcome pattern further clarifies the role of these resources. SRL and ASE were both linked with lower PRO and higher ENG. Baseline PRO and ENG remained significant predictors of T3 PRO and ENG, which shows that the models estimated later functioning while accounting for earlier status. The results therefore support a cautious but meaningful conclusion: LCU influenced later academic functioning through intermediate regulatory and efficacy-related resources above students’ baseline academic functioning.

### 5.4 Interpretation of procrastination and engagement

The results for PRO and ENG should be interpreted with different emphases. For ENG, the positive SRL and ASE paths suggest that learning-centred GenAI use matters because it helps students sustain task involvement through planning, monitoring, and confidence in managing academic demands. The auxiliary evidence also supports this interpretation: ENG correlated with LMS study sessions and teacher engagement ratings.

For PRO, the direct LCU path was not significant after mechanisms and baseline PRO were included. The negative association appeared through SRL and ASE. This means that LCU should not be interpreted as directly reducing procrastination. A student may use GenAI in learning-centred ways, but delay-related difficulties still depend on self-regulation and confidence in managing academic work. The result is theoretically useful because it prevents an overly simple interpretation of GenAI as a direct solution to academic delay.

Considering both outcomes is important. ENG reflects energy, focus, and sustained investment. PRO reflects delay and difficulty initiating or completing tasks. The findings show that these two aspects of academic functioning were connected to the same psychological resources, but the interpretation is not identical. LCU was linked to both outcomes through SRL and ASE, while baseline outcome levels remained important.

### 5.5 Instructional implications

The findings suggest that educational guidance should focus on how students use GenAI during academic work. The issue is not only whether students use GenAI or how often they use it. The more relevant question is whether students use it to clarify tasks, organise steps, verify outputs, revise their own work, and retain responsibility for academic judgement.

One practical implication is to make students’ GenAI-supported learning process visible. In selected assignments, instructors could ask students to submit a brief AI revision-trail report. Such a report might document: the task difficulty that prompted GenAI use; the way GenAI was used to clarify requirements, compare routes, or generate alternatives; how the student checked, modified, or rejected AI output; and what final judgement remained the student’s own. This design aligns closely with the LCU measure and with the auxiliary revision-trail evidence in the present dataset.

The results also support teacher guidance that distinguishes learning-centred use from answer-oriented reliance. Guidance can focus on verification, revision, and explanation. It can also ask students to compare AI-generated responses with course materials, explain why they accepted or rejected suggestions, and demonstrate independent understanding in follow-up activities. These practices keep SRL and ASE at the centre of GenAI-supported academic work.

These practices may be especially important for novice users, who may mistake fluent AI output for reliable understanding. A practical scaffold is to require students to separate three steps: first, state what they asked GenAI to help with; second, identify what they checked against course materials or other sources; and third, explain what they changed, rejected, or kept in the final work. Instructors can also use low-stakes exercises in which students compare an AI-generated explanation with a course-based answer, identify omissions or errors, and revise the explanation in their own words. Such activities reduce the risk that GenAI produces only surface-level fluency or inflated confidence, while keeping responsibility for judgement and revision with the learner.

### 5.6 Limitations and future research

Several limitations should be noted. First, although the study used a multi-wave design with baseline controls, it remains observational. The findings support time-ordered predictive pathway effects, but they do not establish experimental causal effects. The outcomes were academic procrastination and learning engagement, so the study should not be presented as evidence that GenAI use improves academic achievement or learning ability. Claims about achievement gains would require repeated grade, assignment-performance, or task-performance data.

Second, panel retention was selective. Students retained through T3 reported higher LCU, baseline SRL, baseline ENG, GenAI-user status, and GPA band, and lower baseline PRO. The IPW models produced the same substantive pattern, which reduces concern that the main pathways were driven only by observed retention differences. Even so, the complete-case sample may over-represent students who were already more academically engaged, more self-regulated, and more comfortable using GenAI. The findings should therefore be generalised with caution to students with weaker academic engagement, lower prior achievement, limited GenAI experience, or unobserved reasons for dropout.

Third, although SRL and ASE were separated into T2a and T2b modules, the interval between them was short. This separation reduces same-occasion measurement and provides a more plausible ordering than a single-wave mechanism model, but it is not sufficient to establish long-term development in efficacy beliefs. The SRL-to-ASE path should therefore be interpreted as a temporally separated association consistent with the theory-specified process, not as evidence of enduring causal change.

Fourth, the focal measures were brief and context adapted. Although internal consistency and CR were generally acceptable, several AVE values were below the conventional 0.50 threshold. This should be regarded as a measurement limitation, especially for claims about convergent validity. The LCU and AOR measures are useful for the present process model, but they should not yet be treated as fully validated scales. Future studies should examine their item structure, convergent validity, and discriminant validity in independent samples and across different institutional and disciplinary settings.

Fifth, the auxiliary behavioural and rater indicators were available for subsamples and were used as convergent evidence, not as replacements for the main outcomes. Prompt-log, revision-trail, LMS, and teacher-rating indicators may be affected by consent, course context, and data-availability selection. Future research should incorporate fuller prompt logs, learning-management-system traces, teacher ratings, assignment scores, and task-based assessments using designs in which behavioural measures are collected systematically from the outset.

Finally, the heterogeneous disciplinary and year-level composition of the sample should be interpreted cautiously. Discipline and year level were included as covariates, but the present study was not designed to test whether the LCU–SRL/ASE–functioning pathway differs across fields of study or stages of university education. Such differences are plausible. For example, students in writing-intensive, programming-intensive, or clinically oriented fields may encounter different opportunities and risks when using GenAI. Future studies should examine whether discipline, year level, and assessment type moderate the psychological pathways tested here.

## 6 Conclusion

This study examined whether university students’ learning-centred use of GenAI in academic work affected later academic functioning through SRL and ASE. The findings indicate that the learning-centred quality of GenAI use was more informative than simple exposure or frequency. Students who reported stronger LCU at T1 also reported higher SRL at T2a and higher ASE at T2b, and these psychological resources predicted lower PRO and higher ENG at T3 after baseline outcomes were controlled.

The results should not be interpreted as evidence that GenAI use directly changes academic functioning or produces within-person change by itself. Direct paths from LCU to T3 PRO and ENG were not statistically significant once baseline outcomes and psychological mechanisms were included. A more precise conclusion is that LCU was linked to later academic functioning mainly through intermediate regulatory and efficacy-related resources.

The educational implication is therefore not simply to encourage or prohibit GenAI use. The more relevant issue is whether students are guided to use GenAI in ways that keep planning, monitoring, verification, revision, and final academic judgement under their own responsibility. Future research should validate the brief LCU and AOR measures, extend the time interval between psychological mechanisms, and combine self-report with fuller behavioural and task-based evidence of GenAI-supported learning. In this sense, the study reframes GenAI use as a learner-regulated academic practice rather than as a simple exposure variable.

## Data Availability

The original contributions presented in the study are included in the article/Supplementary material, further inquiries can be directed to the corresponding author.

## Supplementary material

SUPPLEMENTARY MATERIAL 1 Ethics approval letter.

SUPPLEMENTARY MATERIAL 2 English reporting version of the questionnaire.

SUPPLEMENTARY MATERIAL 3 De-identified minimal analytic dataset.
