# Supplementary material for: Learning-centred use of generative AI and later academic functioning: a baseline-adjusted three-wave panel study
Source: Front Psychol. 2026 Jul 1;17:1878514. doi: 10.3389/fpsyg.2026.1878514 (PMC13370561; doi:10.3389/fpsyg.2026.1878514)
Supplement: SUPPLEMENTARY MATERIAL 1 — Ethics approval letter. [file Data_Sheet_1.PDF]

Sichuan Construction Vocational Education Group

Research Activity Ethics Review Approval

Approval No.: Sichuan Construction Vocational Education Group Research Ethics Review [2026] No. 052

Project Title: Quality of Academic Use of Generative Artificial Intelligence and Subsequent Academic Functioning among Chinese University Students: A Three-Wave Longitudinal Study of Self-Regulated Learning and Academic Self-Efficacy

Principal Investigator: Wei Dai

Based on the research proposal, three-wave questionnaire, participant informed consent documents, and related materials submitted by the applicant, this institution determines that the study is a **minimum-risk questionnaire survey** conducted among students from multiple higher education institutions within Sichuan Province. The research purpose is clear, the overall risk is controllable, and the study meets the basic ethical requirements for research involving human participants. The study is hereby approved for implementation in accordance with the submitted protocol.

This approval is granted under the following conditions:

1. Written or electronic informed consent must be obtained from participants prior to formal participation. Participants must be clearly informed of the research purpose, data usage, confidentiality measures, and their right to withdraw.
2. The study shall not publicly collect or disclose directly identifiable information, including names, student identification numbers, national identification numbers, or telephone numbers. Papers, reports, and shared data must be processed using anonymisation or de-identification.
3. Questionnaire data and related materials shall be used solely for this study and directly related academic purposes. The research team must properly store the data and adopt necessary data security measures.
4. If there are any substantial changes to the research title, participant scope, questionnaire content, recruitment method, informed consent procedures, data processing methods, or publication plans, the study must be resubmitted for ethics review or filing prior to implementation. In the event of participant complaints, data security incidents, or any other circumstances that may affect participants' rights and interests, these must be reported in a timely manner.

This approval shall take effect from the date of issuance and shall remain valid provided that there are no substantial changes to the research content or procedures. When citing the ethics approval in papers, research reports, or related public documents, the research team must accurately use the approval number "Sichuan Construction Vocational Education Group Research Ethics Review [2026] No. 052".

Approving Institution: Sichuan Construction Vocational Education Group

Date: 8 January 2026

(Official Seal)

四川建设职业教育集团

5100016070
