# Supplementary material for: Learning-centred use of generative AI and later academic functioning: a baseline-adjusted three-wave panel study
Source: Front Psychol. 2026 Jul 1;17:1878514. doi: 10.3389/fpsyg.2026.1878514 (PMC13370561; doi:10.3389/fpsyg.2026.1878514)
Supplement: SUPPLEMENTARY MATERIAL 2 — English reporting version of the questionnaire. [file Data_Sheet_2.DOCX]

**Supplementary Material 2: English Survey Questionnaire**

*Learning-Centred Use of Generative AI and Later Academic Functioning*

This appendix provides the English reporting version of the questionnaire. The original survey was administered in Chinese. The survey did not request names, student numbers, national identification numbers, telephone numbers, or other directly identifying information. Wave matching used anonymised respondent IDs.

Unless otherwise stated, focal scale items used a five-point Likert response format: 1 = strongly disagree; 2 = disagree; 3 = neither agree nor disagree; 4 = agree; 5 = strongly agree. Higher scores indicate more of the named construct. Attention-check items are not reproduced here to preserve data-quality procedures.

# A1. Participant information page and consent prompt

| **Element** | **English wording** |
| --- | --- |
| Study purpose | This study examines how university students use generative AI during academic work and how such use is linked to learning processes and later academic functioning. |
| Voluntary participation | Participation is voluntary. You may stop answering the questionnaire at any time without penalty. |
| Confidentiality | The survey does not ask for directly identifying information. Data will be analysed in anonymised or de-identified form. |
| Data use | The information you provide will be used only for this study and directly related academic purposes. |
| Consent prompt | I have read the study information above, understand the voluntary nature of participation, and agree to take part in this anonymous academic survey. |

# A2. Wave structure

| **Wave/module** | **Survey role** | **Content** |
| --- | --- | --- |
| T1 | Baseline survey | Background characteristics, GenAI-use profile, LCU, TGS, AOR, and baseline SRL, ASE, PRO, and ENG. |
| T2a | Mechanism module 1 | Self-regulated learning (SRL). |
| T2b | Mechanism module 2 | Academic self-efficacy (ASE), administered after T2a within the T2 module window. |
| T3 | Outcome survey | Academic procrastination (PRO) and learning engagement (ENG). |
| Auxiliary evidence | Optional or data-available subsamples | Prompt-log records, revision-trail reports, course-platform indicators, and teacher/rater engagement judgements, where consent and data availability permitted. |

# A3. T1 background and GenAI-use profile items

## A3.1 Background characteristics

| **Code** | **Question** | **Response options / coding** |
| --- | --- | --- |
| B1 | Gender | Female; Male; Other / prefer not to say |
| B2 | Student type | Domestic student; International student; Other / not applicable |
| B3 | Year level | Year 1; Year 2; Year 3; Year 4 or above |
| B4 | Broad discipline | Engineering/computer science; management; medicine/health; natural sciences; social sciences; economics; humanities; arts; agriculture; other |
| B5 | Self-reported GPA band | < 2.50; 2.50–2.99; 3.00–3.49; ≥ 3.50; not applicable / prefer not to say |

## A3.2 GenAI-use profile

| **Code** | **Question** | **Response options / coding** |
| --- | --- | --- |
| G1 | Have you previously used any generative AI tool for academic or non-academic purposes? | No; Yes |
| G2 | How often have you used generative AI during the past 30 days? | Five-point ordinal frequency scale, from never/almost never to daily or almost daily |
| G3 | Which generative AI tool have you used most often recently? | DeepSeek; Doubao; ChatGPT; Kimi; ERNIE Bot / Wenxin Yiyan; Tongyi Qianwen; other; none |
| G4 | In which situations have you used generative AI? Please select all that apply. | Learning or research; life assistance; practical tasks such as projects, presentations, code or data analysis; emotional support; other |

# A4. Core scale items

All items in this section used the five-point Likert response format unless otherwise stated. Composite scores were calculated as item means when sufficient item responses were available.

## A4.1 T1 focal GenAI-use and contextual items

| **Item code** | **Construct** | **English item wording** |
| --- | --- | --- |
| LCU1_T1 | Learning-centred use of GenAI in academic work | I use GenAI to help explain difficult course concepts. |
| LCU2_T1 | Learning-centred use of GenAI in academic work | Before starting academic tasks, I use GenAI to organise ideas or steps. |
| LCU3_T1 | Learning-centred use of GenAI in academic work | I use GenAI to reorganise learning materials so that they are easier to understand. |
| LCU4_T1 | Learning-centred use of GenAI in academic work | When I get stuck, I ask GenAI for hints or alternative approaches rather than a finished answer. |
| LCU5_T1 | Learning-centred use of GenAI in academic work | Before submitting assignments, I use GenAI to check expression, logic, or possible problems. |
| LCU6_T1 | Learning-centred use of GenAI in academic work | I treat GenAI as an auxiliary tool in academic tasks, not as the final judge. |
| TGS1_T1 | Teacher guidance support | Teachers explain in which learning situations AI use is appropriate. |
| TGS2_T1 | Teacher guidance support | Teachers remind us to verify the accuracy of AI outputs. |
| TGS3_T1 | Teacher guidance support | Course tasks require a combination of AI assistance and students’ independent judgement. |
| TGS4_T1 | Teacher guidance support | Teachers give concrete feedback on how we use AI in academic work. |
| AOR1_T1 | Answer-oriented reliance | I sometimes expect AI to provide an answer that can be used directly. |
| AOR2_T1 | Answer-oriented reliance | When time is limited, I tend to rely on AI to complete tasks quickly. |
| AOR3_T1 | Answer-oriented reliance | If an AI output looks reasonable, I may not check it carefully. |
| AOR4_T1 | Answer-oriented reliance | I sometimes rely on AI instead of thinking through the task myself. |

## A4.2 T1 baseline psychological resources and academic functioning

| **Item code** | **Construct** | **English item wording** |
| --- | --- | --- |
| SRL1_T1base | Self-regulated learning baseline | I set clear goals for academic tasks. |
| SRL2_T1base | Self-regulated learning baseline | I usually plan the steps for completing academic tasks in advance. |
| SRL3_T1base | Self-regulated learning baseline | During learning, I check whether I really understand. |
| SRL4_T1base | Self-regulated learning baseline | If a learning strategy is not working, I adjust it. |
| SRL5_T1base | Self-regulated learning baseline | I check my progress before deadlines. |
| SRL6_T1base | Self-regulated learning baseline | Even when tasks are difficult, I try to persist. |
| ASE1_T1base | Academic self-efficacy baseline | I am confident that I can master challenging course materials. |
| ASE2_T1base | Academic self-efficacy baseline | I believe I can solve complex course assignments. |
| ASE3_T1base | Academic self-efficacy baseline | Even under pressure, I can meet course requirements. |
| ASE4_T1base | Academic self-efficacy baseline | I can handle most academic tasks independently. |
| ASE5_T1base | Academic self-efficacy baseline | With appropriate methods, I can achieve the learning results I expect. |
| PRO1_T1base | Academic procrastination baseline | I often delay starting important academic tasks. |
| PRO2_T1base | Academic procrastination baseline | Even when I know coursework takes time, I postpone it. |
| PRO3_T1base | Academic procrastination baseline | I often wait until deadlines are close before seriously working on academic tasks. |
| PRO4_T1base | Academic procrastination baseline | I often leave reviewing or preparation until the last moment. |
| PRO5_T1base | Academic procrastination baseline | When learning tasks become difficult, I easily turn to other things instead of continuing. |
| PRO6_T1base | Academic procrastination baseline | Without external pressure, I find it difficult to complete academic work on time. |
| ENG1_T1base | Learning engagement baseline | Recently, I usually feel energetic when learning. |
| ENG2_T1base | Learning engagement baseline | I can focus my attention on current learning tasks. |
| ENG3_T1base | Learning engagement baseline | When learning, I often become immersed in the task. |
| ENG4_T1base | Learning engagement baseline | I am willing to invest sustained effort in current learning tasks. |

## A4.3 T2a self-regulated learning module

| **Item code** | **Construct** | **English item wording** |
| --- | --- | --- |
| SRL1_T2a | Self-regulated learning | I set clear goals for academic tasks. |
| SRL2_T2a | Self-regulated learning | I usually plan the steps for completing academic tasks in advance. |
| SRL3_T2a | Self-regulated learning | During learning, I check whether I really understand. |
| SRL4_T2a | Self-regulated learning | If a learning strategy is not working, I adjust it. |
| SRL5_T2a | Self-regulated learning | I check my progress before deadlines. |
| SRL6_T2a | Self-regulated learning | Even when tasks are difficult, I try to persist. |

## A4.4 T2b academic self-efficacy module

| **Item code** | **Construct** | **English item wording** |
| --- | --- | --- |
| ASE1_T2b | Academic self-efficacy | I am confident that I can master challenging course materials. |
| ASE2_T2b | Academic self-efficacy | I believe I can solve complex course assignments. |
| ASE3_T2b | Academic self-efficacy | Even under pressure, I can meet course requirements. |
| ASE4_T2b | Academic self-efficacy | I can handle most academic tasks independently. |
| ASE5_T2b | Academic self-efficacy | With appropriate methods, I can achieve the learning results I expect. |

## A4.5 T3 academic functioning outcomes

| **Item code** | **Construct** | **English item wording** |
| --- | --- | --- |
| PRO1_T3 | Academic procrastination | I often delay starting important academic tasks. |
| PRO2_T3 | Academic procrastination | Even when I know coursework takes time, I postpone it. |
| PRO3_T3 | Academic procrastination | I often wait until deadlines are close before seriously working on academic tasks. |
| PRO4_T3 | Academic procrastination | I often leave reviewing or preparation until the last moment. |
| PRO5_T3 | Academic procrastination | When learning tasks become difficult, I easily turn to other things instead of continuing. |
| PRO6_T3 | Academic procrastination | Without external pressure, I find it difficult to complete academic work on time. |
| ENG1_T3 | Learning engagement | Recently, I usually feel energetic when learning. |
| ENG2_T3 | Learning engagement | I can focus my attention on current learning tasks. |
| ENG3_T3 | Learning engagement | When learning, I often become immersed in the task. |
| ENG4_T3 | Learning engagement | I am willing to invest sustained effort in current learning tasks. |

# A5. Optional auxiliary materials used as convergent evidence

The following materials were not used as primary outcomes. They were available only for consenting or data-available subsamples and were used to examine whether self-report measures aligned with behaviourally adjacent evidence.

| **Code** | **Auxiliary material** | **English wording / operational definition** |
| --- | --- | --- |
| AUX1 | Optional prompt-log record | Students who consented could provide anonymised examples or counts of academic GenAI prompts. The analytic indicator was the proportion of prompts involving checking, verification, revision, or evaluation rather than simple answer generation. |
| AUX2 | Revision-trail report prompt 1 | What academic difficulty or task requirement prompted you to consult GenAI? |
| AUX3 | Revision-trail report prompt 2 | How did you use GenAI to clarify requirements, organise steps, compare alternatives, or generate possible approaches? |
| AUX4 | Revision-trail report prompt 3 | How did you check, modify, reject, or revise the AI output? |
| AUX5 | Revision-trail report prompt 4 | What final academic judgement remained your own? |
| AUX6 | Course-platform indicators | Where course-level permission and anonymised records were available, late submission count, on-time submission rate, and learning-management-system study sessions were used as convergent evidence. |
| AUX7 | Teacher/rater engagement judgement | Where course-level permission and anonymised ratings were available, external engagement ratings were used only as convergent evidence and not as primary outcomes. |

# A6. Scale scoring summary

| **Construct** | **Wave/module** | **Items** | **Scoring** |
| --- | --- | --- | --- |
| LCU | T1 | 6 | Mean of LCU1–LCU6; higher = more learning-centred use |
| TGS | T1 | 4 | Mean of TGS1–TGS4; higher = more teacher guidance support |
| AOR | T1 | 4 | Mean of AOR1–AOR4; higher = more answer-oriented reliance |
| SRL baseline | T1 baseline | 6 | Mean of SRL1–SRL6 at baseline |
| SRL | T2a | 6 | Mean of SRL1–SRL6 at T2a |
| ASE baseline | T1 baseline | 5 | Mean of ASE1–ASE5 at baseline |
| ASE | T2b | 5 | Mean of ASE1–ASE5 at T2b |
| PRO baseline | T1 baseline | 6 | Mean of PRO1–PRO6 at baseline |
| PRO | T3 | 6 | Mean of PRO1–PRO6 at T3 |
| ENG baseline | T1 baseline | 4 | Mean of ENG1–ENG4 at baseline |
| ENG | T3 | 4 | Mean of ENG1–ENG4 at T3 |

# A7. Notes for journal submission

| **Note** | **Details** |
| --- | --- |
| Translation status | This is an English reporting version of the instrument. The administered questionnaire was in Chinese and used equivalent item meanings. |
| Data protection | The questionnaire did not collect direct identifiers. Wave matching used anonymised respondent IDs. |
| Interpretation of scales | Higher values indicate more of the named construct. No reverse-coded focal items are reported in this appendix. |
| Use in manuscript | The appendix supports the reporting of item wording, scale scoring, and wave structure. Measurement diagnostics are reported in the Supplementary Tables. |
