# Supplementary material for: Learning-centred use of generative AI and later academic functioning: a baseline-adjusted three-wave panel study
Source: Front Psychol. 2026 Jul 1;17:1878514. doi: 10.3389/fpsyg.2026.1878514 (PMC13370561; doi:10.3389/fpsyg.2026.1878514)
Supplement: Supplementary file 4 [file Data_Sheet_4.DOCX]

**Supplementary Tables**

Learning-Centred Use of Generative AI and Later Academic Functioning: A Baseline-Adjusted Three-Wave Panel Study

**Supplementary Table S1. Summary of measurement diagnostics for the focal measures.**

| **Construct** | **Wave/module** | **Items** | **Complete-item N** | **α** | **Loading range** | **CR** | **AVE** | **Interpretation** |
| --- | --- | --- | --- | --- | --- | --- | --- | --- |
| LCU | T1 | 6 | 1,162 | 0.872 | 0.716–0.737 | 0.873 | 0.533 | AVE ≥ 0.50 |
| TGS | T1 | 4 | 1,159 | 0.730 | 0.602–0.700 | 0.731 | 0.406 | AVE < 0.50 |
| AOR | T1 | 4 | 1,163 | 0.698 | 0.585–0.637 | 0.700 | 0.368 | AVE < 0.50 |
| SRL baseline | T1 baseline | 6 | 1,133 | 0.815 | 0.622–0.673 | 0.818 | 0.428 | AVE < 0.50 |
| ASE baseline | T1 baseline | 5 | 1,150 | 0.760 | 0.603–0.652 | 0.766 | 0.396 | AVE < 0.50 |
| PRO baseline | T1 baseline | 6 | 1,146 | 0.791 | 0.594–0.654 | 0.795 | 0.392 | AVE < 0.50 |
| ENG baseline | T1 baseline | 4 | 1,172 | 0.747 | 0.642–0.661 | 0.747 | 0.425 | AVE < 0.50 |
| SRL | T2a | 6 | 928 | 0.811 | 0.595–0.715 | 0.813 | 0.422 | AVE < 0.50 |
| ASE | T2b | 5 | 943 | 0.752 | 0.559–0.642 | 0.754 | 0.380 | AVE < 0.50 |
| PRO | T3 | 6 | 741 | 0.796 | 0.607–0.672 | 0.800 | 0.400 | AVE < 0.50 |
| ENG | T3 | 4 | 759 | 0.740 | 0.618–0.684 | 0.746 | 0.423 | AVE < 0.50 |

*Note.* α = Cronbach’s alpha; CR = composite reliability; AVE = average variance extracted. Loading range refers to the range of standardised item loadings within each measure. Complete-item N indicates the number of respondents with valid responses on all items of the corresponding measure.

**Supplementary Table S2. Item-level standardised factor loadings for the focal measures.**

| **Construct** | **Wave/module** | **Item code** | **English wording** | **Standardised loading** | |
| --- | --- | --- | --- | --- | --- |
| LCU | T1 | LCU1_T1 | I use GenAI to help explain difficult course concepts. | | 0.725 |
| LCU | T1 | LCU2_T1 | Before starting academic tasks, I use GenAI to organise ideas or steps. | | 0.737 |
| LCU | T1 | LCU3_T1 | I use GenAI to reorganise learning materials so that they are easier to understand. | | 0.736 |
| LCU | T1 | LCU4_T1 | When I get stuck, I ask GenAI for hints or alternative approaches rather than a finished answer. | | 0.729 |
| LCU | T1 | LCU5_T1 | Before submitting assignments, I use GenAI to check expression, logic, or possible problems. | | 0.716 |
| LCU | T1 | LCU6_T1 | I treat GenAI as an auxiliary tool in academic tasks, not as the final judge. | | 0.737 |
| TGS | T1 | TGS1_T1 | Teachers explain in which learning situations AI use is appropriate. | | 0.700 |
| TGS | T1 | TGS2_T1 | Teachers remind us to verify the accuracy of AI outputs. | | 0.622 |
| TGS | T1 | TGS3_T1 | Course tasks require a combination of AI assistance and students’ independent judgement. | | 0.602 |
| TGS | T1 | TGS4_T1 | Teachers give concrete feedback on how we use AI in academic work. | | 0.619 |
| AOR | T1 | AOR1_T1 | I sometimes expect AI to provide an answer that can be used directly. | | 0.601 |
| AOR | T1 | AOR2_T1 | When time is limited, I tend to rely on AI to complete tasks quickly. | | 0.637 |
| AOR | T1 | AOR3_T1 | If an AI output looks reasonable, I may not check it carefully. | | 0.585 |
| AOR | T1 | AOR4_T1 | I sometimes rely on AI instead of thinking through the task myself. | | 0.604 |
| SRL baseline | T1 baseline | SRL1_T1base | I set clear goals for academic tasks. | | 0.622 |
| SRL baseline | T1 baseline | SRL2_T1base | I usually plan the steps for completing academic tasks in advance. | | 0.665 |
| SRL baseline | T1 baseline | SRL3_T1base | During learning, I check whether I really understand. | | 0.669 |
| SRL baseline | T1 baseline | SRL4_T1base | If a learning strategy is not working, I adjust it. | | 0.662 |
| SRL baseline | T1 baseline | SRL5_T1base | I check my progress before deadlines. | | 0.673 |
| SRL baseline | T1 baseline | SRL6_T1base | Even when tasks are difficult, I try to persist. | | 0.635 |
| ASE baseline | T1 baseline | ASE1_T1base | I am confident that I can master challenging course materials. | | 0.612 |
| ASE baseline | T1 baseline | ASE2_T1base | I believe I can solve complex course assignments. | | 0.652 |
| ASE baseline | T1 baseline | ASE3_T1base | Even under pressure, I can meet course requirements. | | 0.644 |
| ASE baseline | T1 baseline | ASE4_T1base | I can handle most academic tasks independently. | | 0.603 |
| ASE baseline | T1 baseline | ASE5_T1base | With appropriate methods, I can achieve the learning results I expect. | | 0.632 |
| PRO baseline | T1 baseline | PRO1_T1base | I often delay starting important academic tasks. | | 0.615 |
| PRO baseline | T1 baseline | PRO2_T1base | Even when I know coursework takes time, I postpone it. | | 0.652 |
| PRO baseline | T1 baseline | PRO3_T1base | I often wait until deadlines are close before seriously working on academic tasks. | | 0.594 |
| PRO baseline | T1 baseline | PRO4_T1base | I often leave reviewing or preparation until the last moment. | | 0.609 |
| PRO baseline | T1 baseline | PRO5_T1base | When learning tasks become difficult, I easily turn to other things instead of continuing. | | 0.632 |
| PRO baseline | T1 baseline | PRO6_T1base | Without external pressure, I find it difficult to complete academic work on time. | | 0.654 |
| ENG baseline | T1 baseline | ENG1_T1base | Recently, I usually feel energetic when learning. | | 0.649 |
| ENG baseline | T1 baseline | ENG2_T1base | I can focus my attention on current learning tasks. | | 0.655 |
| ENG baseline | T1 baseline | ENG3_T1base | When learning, I often become immersed in the task. | | 0.642 |
| ENG baseline | T1 baseline | ENG4_T1base | I am willing to invest sustained effort in current learning tasks. | | 0.661 |
| SRL | T2a | SRL1_T2a | I set clear goals for academic tasks. | | 0.595 |
| SRL | T2a | SRL2_T2a | I usually plan the steps for completing academic tasks in advance. | | 0.642 |
| SRL | T2a | SRL3_T2a | During learning, I check whether I really understand. | | 0.673 |
| SRL | T2a | SRL4_T2a | If a learning strategy is not working, I adjust it. | | 0.622 |
| SRL | T2a | SRL5_T2a | I check my progress before deadlines. | | 0.644 |
| SRL | T2a | SRL6_T2a | Even when tasks are difficult, I try to persist. | | 0.715 |
| ASE | T2b | ASE1_T2b | I am confident that I can master challenging course materials. | | 0.637 |
| ASE | T2b | ASE2_T2b | I believe I can solve complex course assignments. | | 0.642 |
| ASE | T2b | ASE3_T2b | Even under pressure, I can meet course requirements. | | 0.559 |
| ASE | T2b | ASE4_T2b | I can handle most academic tasks independently. | | 0.619 |
| ASE | T2b | ASE5_T2b | With appropriate methods, I can achieve the learning results I expect. | | 0.623 |
| PRO | T3 | PRO1_T3 | I often delay starting important academic tasks. | | 0.620 |
| PRO | T3 | PRO2_T3 | Even when I know coursework takes time, I postpone it. | | 0.672 |
| PRO | T3 | PRO3_T3 | I often wait until deadlines are close before seriously working on academic tasks. | | 0.627 |
| PRO | T3 | PRO4_T3 | I often leave reviewing or preparation until the last moment. | | 0.646 |
| PRO | T3 | PRO5_T3 | When learning tasks become difficult, I easily turn to other things instead of continuing. | | 0.621 |
| PRO | T3 | PRO6_T3 | Without external pressure, I find it difficult to complete academic work on time. | | 0.607 |
| ENG | T3 | ENG1_T3 | Recently, I usually feel energetic when learning. | | 0.618 |
| ENG | T3 | ENG2_T3 | I can focus my attention on current learning tasks. | | 0.684 |
| ENG | T3 | ENG3_T3 | When learning, I often become immersed in the task. | | 0.647 |
| ENG | T3 | ENG4_T3 | I am willing to invest sustained effort in current learning tasks. | | 0.650 |

*Note.* Standardised loadings were estimated from one-factor item models for each focal measure. The item wording corresponds to the English reporting version in Supplementary Material 2.

**Supplementary Table S3. Full dummy-coded covariate coefficients for the baseline-adjusted path models.**

| **Outcome** | **Predictor** | **β** | **SE** | **t** | **p** | **95% CI** |
| --- | --- | --- | --- | --- | --- | --- |
| SRL T2a | gender: Male | 0.036 | 0.064 | 0.572 | 0.567 | [−0.088, 0.161] |
| SRL T2a | gender: Another/prefer not to say | -0.216 | 0.170 | -1.273 | 0.203 | [−0.548, 0.117] |
| SRL T2a | student type: International | -0.108 | 0.150 | -0.718 | 0.473 | [−0.403, 0.187] |
| SRL T2a | year level: Year 2 | 0.012 | 0.087 | 0.137 | 0.891 | [−0.160, 0.183] |
| SRL T2a | year level: Year 3 | 0.079 | 0.086 | 0.920 | 0.358 | [−0.089, 0.248] |
| SRL T2a | year level: Year 4 or above | -0.037 | 0.087 | -0.431 | 0.666 | [−0.207, 0.132] |
| SRL T2a | discipline: Agriculture | -0.260 | 0.216 | -1.207 | 0.227 | [−0.683, 0.162] |
| SRL T2a | discipline: Arts | 0.019 | 0.160 | 0.120 | 0.905 | [−0.295, 0.334] |
| SRL T2a | discipline: Economics | -0.102 | 0.155 | -0.656 | 0.512 | [−0.406, 0.202] |
| SRL T2a | discipline: Humanities | -0.006 | 0.134 | -0.045 | 0.964 | [−0.268, 0.256] |
| SRL T2a | discipline: Management | -0.180 | 0.091 | -1.977 | 0.048 | [−0.359, −0.002] |
| SRL T2a | discipline: Medicine and health | -0.096 | 0.099 | -0.970 | 0.332 | [−0.291, 0.098] |
| SRL T2a | discipline: Natural sciences | -0.172 | 0.121 | -1.420 | 0.156 | [−0.410, 0.065] |
| SRL T2a | discipline: Other | -0.195 | 0.207 | -0.944 | 0.345 | [−0.600, 0.210] |
| SRL T2a | discipline: Social sciences | -0.213 | 0.118 | -1.796 | 0.072 | [−0.445, 0.019] |
| ASE T2b | gender: Male | 0.089 | 0.064 | 1.381 | 0.167 | [−0.037, 0.215] |
| ASE T2b | gender: Another/prefer not to say | -0.314 | 0.225 | -1.398 | 0.162 | [−0.755, 0.126] |
| ASE T2b | student type: International | -0.051 | 0.151 | -0.334 | 0.738 | [−0.348, 0.246] |
| ASE T2b | year level: Year 2 | 0.020 | 0.085 | 0.229 | 0.819 | [−0.148, 0.187] |
| ASE T2b | year level: Year 3 | -0.001 | 0.090 | -0.014 | 0.988 | [−0.178, 0.175] |
| ASE T2b | year level: Year 4 or above | -0.071 | 0.088 | -0.800 | 0.424 | [−0.244, 0.102] |
| ASE T2b | discipline: Agriculture | 0.265 | 0.217 | 1.218 | 0.223 | [−0.161, 0.691] |
| ASE T2b | discipline: Arts | 0.159 | 0.160 | 0.994 | 0.320 | [−0.154, 0.471] |
| ASE T2b | discipline: Economics | 0.169 | 0.131 | 1.287 | 0.198 | [−0.088, 0.426] |
| ASE T2b | discipline: Humanities | -0.085 | 0.138 | -0.612 | 0.541 | [−0.355, 0.186] |
| ASE T2b | discipline: Management | 0.083 | 0.094 | 0.888 | 0.374 | [−0.100, 0.267] |
| ASE T2b | discipline: Medicine and health | 0.128 | 0.114 | 1.117 | 0.264 | [−0.096, 0.352] |
| ASE T2b | discipline: Natural sciences | -0.014 | 0.113 | -0.122 | 0.903 | [−0.234, 0.207] |
| ASE T2b | discipline: Other | 0.120 | 0.248 | 0.486 | 0.627 | [−0.366, 0.607] |
| ASE T2b | discipline: Social sciences | 0.168 | 0.120 | 1.402 | 0.161 | [−0.067, 0.402] |
| PRO T3 | gender: Male | 0.127 | 0.061 | 2.077 | 0.038 | [0.007, 0.246] |
| PRO T3 | gender: Another/prefer not to say | 0.236 | 0.199 | 1.186 | 0.236 | [−0.154, 0.626] |
| PRO T3 | student type: International | -0.091 | 0.152 | -0.600 | 0.549 | [−0.388, 0.206] |
| PRO T3 | year level: Year 2 | 0.009 | 0.081 | 0.115 | 0.909 | [−0.150, 0.169] |
| PRO T3 | year level: Year 3 | -0.030 | 0.087 | -0.349 | 0.727 | [−0.202, 0.141] |
| PRO T3 | year level: Year 4 or above | 0.025 | 0.091 | 0.279 | 0.780 | [−0.153, 0.204] |
| PRO T3 | discipline: Agriculture | -0.225 | 0.202 | -1.110 | 0.267 | [−0.622, 0.172] |
| PRO T3 | discipline: Arts | -0.075 | 0.144 | -0.521 | 0.602 | [−0.358, 0.208] |
| PRO T3 | discipline: Economics | -0.077 | 0.140 | -0.549 | 0.583 | [−0.352, 0.198] |
| PRO T3 | discipline: Humanities | -0.019 | 0.123 | -0.153 | 0.878 | [−0.260, 0.222] |
| PRO T3 | discipline: Management | -0.049 | 0.093 | -0.529 | 0.597 | [−0.230, 0.132] |
| PRO T3 | discipline: Medicine and health | 0.007 | 0.105 | 0.071 | 0.944 | [−0.199, 0.214] |
| PRO T3 | discipline: Natural sciences | -0.077 | 0.100 | -0.766 | 0.443 | [−0.273, 0.120] |
| PRO T3 | discipline: Other | 0.137 | 0.220 | 0.622 | 0.534 | [−0.294, 0.567] |
| PRO T3 | discipline: Social sciences | -0.112 | 0.117 | -0.959 | 0.338 | [−0.341, 0.117] |
| ENG T3 | gender: Male | -0.080 | 0.061 | -1.302 | 0.193 | [−0.199, 0.040] |
| ENG T3 | gender: Another/prefer not to say | -0.190 | 0.258 | -0.736 | 0.462 | [−0.697, 0.316] |
| ENG T3 | student type: International | 0.255 | 0.183 | 1.388 | 0.165 | [−0.105, 0.614] |
| ENG T3 | year level: Year 2 | -0.021 | 0.082 | -0.253 | 0.801 | [−0.181, 0.140] |
| ENG T3 | year level: Year 3 | -0.002 | 0.084 | -0.028 | 0.978 | [−0.168, 0.163] |
| ENG T3 | year level: Year 4 or above | -0.016 | 0.090 | -0.182 | 0.856 | [−0.192, 0.160] |
| ENG T3 | discipline: Agriculture | -0.029 | 0.187 | -0.155 | 0.877 | [−0.395, 0.338] |
| ENG T3 | discipline: Arts | -0.039 | 0.160 | -0.244 | 0.807 | [−0.352, 0.274] |
| ENG T3 | discipline: Economics | -0.019 | 0.129 | -0.149 | 0.882 | [−0.272, 0.234] |
| ENG T3 | discipline: Humanities | -0.134 | 0.130 | -1.033 | 0.302 | [−0.390, 0.121] |
| ENG T3 | discipline: Management | 0.136 | 0.085 | 1.603 | 0.109 | [−0.030, 0.302] |
| ENG T3 | discipline: Medicine and health | -0.092 | 0.121 | -0.755 | 0.450 | [−0.330, 0.146] |
| ENG T3 | discipline: Natural sciences | 0.073 | 0.112 | 0.648 | 0.517 | [−0.147, 0.292] |
| ENG T3 | discipline: Other | 0.155 | 0.269 | 0.575 | 0.565 | [−0.373, 0.682] |
| ENG T3 | discipline: Social sciences | -0.120 | 0.111 | -1.077 | 0.281 | [−0.338, 0.098] |

*Note.* Models used standardised composite scores and HC3 standard errors. The reference groups were female, domestic student, Year 1, and engineering/computer science. Key continuous/ordinal controls are reported in Table 2; this table reports the dummy-coded demographic and discipline covariates.

**Supplementary Table S4. Discriminant-validity diagnostics for the primary focal constructs.**

*Panel A. Fornell-Larcker comparisons based on complete-case composite scores.*

| **Construct** | **1 LCU** | **2 TGS** | **3 AOR** | **4 SRL** | **5 ASE** | **6 PRO** | **7 ENG** |
| --- | --- | --- | --- | --- | --- | --- | --- |
| 1. LCU | **0.730** |  |  |  |  |  |  |
| 2. TGS | 0.283 | **0.637** |  |  |  |  |  |
| 3. AOR | -0.029 | -0.063 | **0.607** |  |  |  |  |
| 4. SRL | 0.431 | 0.267 | -0.027 | **0.650** |  |  |  |
| 5. ASE | 0.309 | 0.237 | -0.004 | 0.375 | **0.616** |  |  |
| 6. PRO | -0.215 | -0.118 | 0.068 | -0.407 | -0.371 | **0.632** |  |
| 7. ENG | 0.270 | 0.215 | -0.061 | 0.471 | 0.305 | -0.300 | **0.650** |

*Panel B. Summary of HTMT diagnostics.*

| **Diagnostic** | **Construct pair** | **Value** | **Interpretation** |
| --- | --- | --- | --- |
| Largest observed HTMT ratio | SRL-ENG | 0.616 | Below 0.85 |
| LCU-SRL HTMT ratio | LCU-SRL | 0.518 | Below 0.85 |

Note. In Panel A, diagonal values in bold are the square roots of AVE values reported in Supplementary Table S1; lower-triangle values are Pearson correlations among composite scores in the complete-case analytic sample (N = 788). For each construct, the square root of AVE exceeded its largest absolute inter-construct correlation. Panel B summarises the HTMT diagnostics reported for the focal measures. LCU = learning-centred use of GenAI in academic work; TGS = teacher guidance support; AOR = answer-oriented reliance; SRL = self-regulated learning; ASE = academic self-efficacy; PRO = academic procrastination; ENG = learning engagement. These diagnostics support empirical separation among the primary focal constructs, but they should be interpreted together with the limitation that several brief, context-adapted measures had AVE values below 0.50.
